# Methanotrophs and methane oxidation in cold wetlands: distribution, ecology, and mechanisms

**DOI:** 10.1093/ismeco/ycag214

**Published:** 2026-07-26

**Authors:** Lerui Zhou, Juanli Yun, Yongcui Deng, Kai Xue, Muhammad Farhan Ul Haque, Andrew Crombie, Anzhou Ma, Yanfen Wang

**Affiliations:** College of Environmental Science and Technology, Shaanxi University of Science and Technology, Xi’an 710021, China; College of Environmental Science and Technology, Shaanxi University of Science and Technology, Xi’an 710021, China; School of Geography, Nanjing Normal University, Nanjing 210023, China; College of Resources and Environment, University of Chinese Academy of Sciences, Beijing 100049, China; School of Biological Sciences, University of the Punjab, Quaid-i-Azam Campus, Lahore 54590, Pakistan; School of Biological Sciences, University of East Anglia, Norwich NR4 7TJ, United Kingdom; College of Resources and Environment, University of Chinese Academy of Sciences, Beijing 100049, China; Research Center for Eco-Environmental Sciences, Chinese Academy of Sciences, Beijing 100085, China; College of Resources and Environment, University of Chinese Academy of Sciences, Beijing 100049, China

**Keywords:** methanotrophs, cold wetlands, microbial biogeochemistry, methane oxidation mechanisms

## Abstract

Wetlands in cold regions are significant natural sources of methane. Methane-oxidizing microbes (methanotrophs) serve as key players in mitigating methane in these ecosystems. A substantial proportion of methane, often the majority, is oxidized by aerobic methane-oxidizing bacteria (aerobic methanotrophs) before being emitted to the atmosphere. Their diversity, environmental distribution, methane oxidation capacity, and metabolic mechanisms have been intensively studied worldwide across diverse ecosystems. The relatively recent discovery of anaerobic methanotrophic archaea (ANME) and “*Candidatus* Methylomirabilota” (NC10 bacteria) revolutionized our understanding of methane cycling, revealing previously unrecognized anaerobic oxidation strategies, often coupled with various environmental available electron acceptors. Here, we review the role of methanotrophs in cold wetlands on a global scale, focusing on recent developments in our knowledge of their ecological distribution, methane oxidation potential, and cold-adaptation strategies. We discuss future research directions for methanotrophs in cold wetlands. Understanding the cold-adapted lifestyle of methanotrophs will necessitate combined efforts in microbiology, biochemistry, and computational biology. Harnessing the power of cold-adapted methanotrophs has the potential to significantly mitigate global warming by enhancing greenhouse gas uptake.

## Introduction

Methane is a potent greenhouse gas (GHG) that contributes ~30% to global warming, with a global warming potential ~28–36 times greater than that of CO_2_ over a 100-year timescale [1–3]. Among natural sources, wetlands are the largest emitters of methane [4], accounting for nearly one-third of total global methane emissions [5, 6].

Regions of the planet, which experience periodic or permanent cold conditions, including the polar zones, high-altitude plateaus, glacial areas, extensive permafrost, and seasonally snow-covered regions [7–11], constitute ~80% of the Earth’s biosphere [12–14], and now face unprecedented challenges because of climate change [15, 16]. According to the Ramsar List (The List of Wetlands of International Importance, 2024), over 40% (over 1100 in 2524) of these wetlands are located in high-latitude regions (above or below 40°) and polar areas [17, 18]. However, this number remains underestimated due to the non-exhaustiveness of the list [17, 19]. We define wetlands in cold regions as cold wetlands, and they represent an important ecosystem in global biogeochemistry cycling.

**Figure 1:**
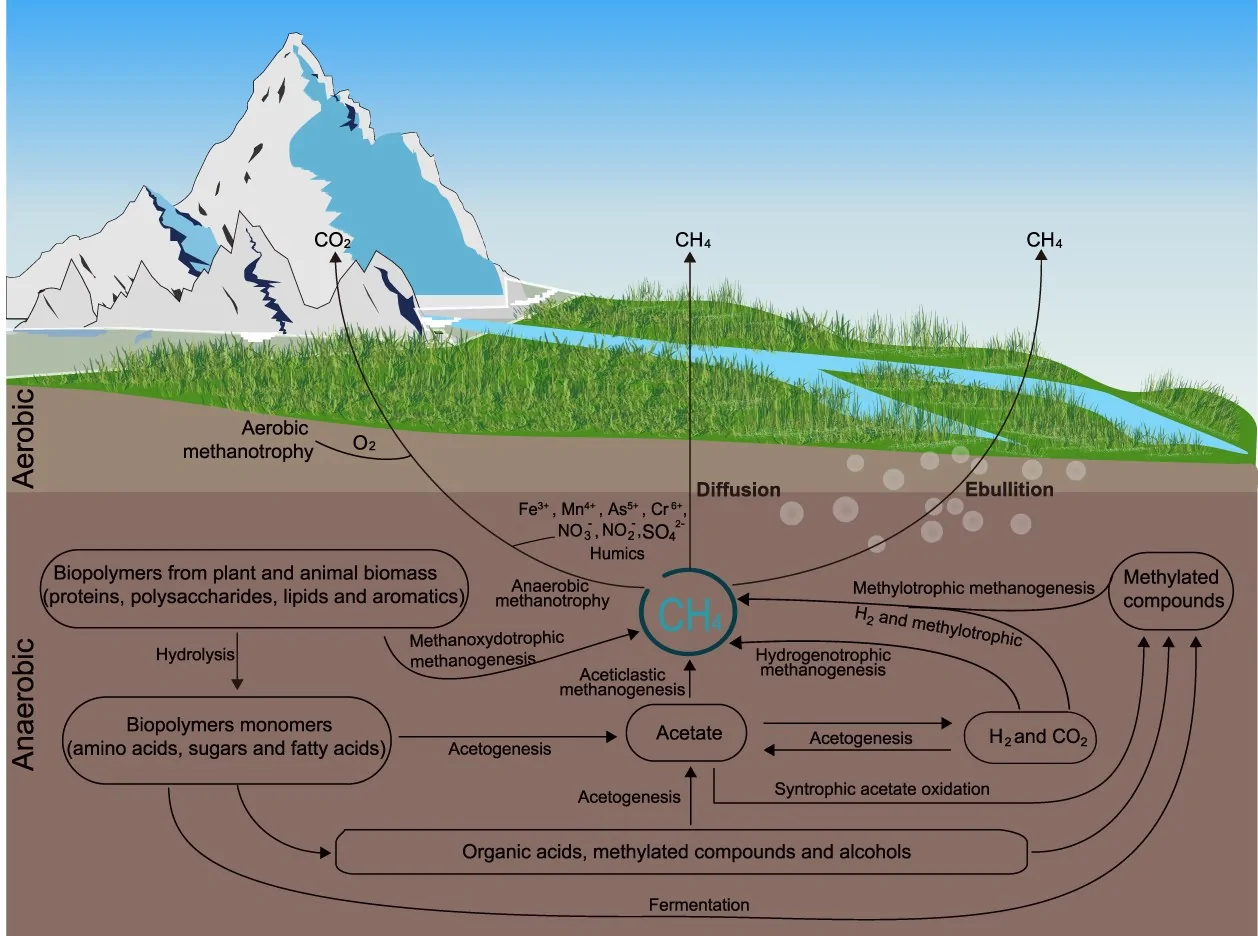
Schematic diagram of microbial methane metabolic processes in cold ecosystems.

Methane generation in cold wetlands is a microbially mediated process involving both bacteria and archaea [20]. Large polymeric organic molecules, such as polysaccharides, lipids, and proteins, which have accumulated over long periods, undergo sequential microbial degradation. Initially, hydrolytic and fermentative microbes decompose these polymers into smaller intermediates, producing compounds such as acetate, methylated compounds, hydrogen, and carbon dioxide. These substrates are subsequently utilized by methanogenic archaea through distinct methanogenic pathways to generate methane [21]. The methane is transported to the surface either by diffusion or ebullition (bubble flux) (Fig. 1).

However, a substantial amount (60% ~ 90%) of this methane does not reach the atmosphere because it is oxidized to carbon dioxide by methane-oxidizing microorganisms (methanotrophs) thereby mitigating methane emissions and reducing the overall greenhouse effect [22–25]. Cold environments host various psychrophilic and psychrotolerant microorganisms that play crucial roles in these oxidation processes [14, 21  26, 27]. The majority of methane consumed in cold wetlands is mediated by aerobic methanotrophs [28], while anaerobic methanotrophs make a comparatively smaller contribution [29].

Methanotrophs utilize methane as their main source of carbon and energy [30]. Aerobic methanotrophs, all belonging to the domain Bacteria, were historically thought to require oxygen as the terminal electron acceptor. In contrast, anaerobic methane oxidation, mediated by both bacteria and archaea, relies on alternative electron acceptors such as nitrate/nitrite, iron, manganese, sulfate, or organic compounds like humic substances [31].

Currently, the identified aerobic methanotrophs belong to three phyla: *Pseudomonadota* [32], *Verrucomicrobiota* [33], and *Actinomycetota* [34]. Methanotrophs from the *Pseudomonadota* can be divided into type I [35, 36] and type II [37], which belong to the γ-*Proteobacteria* and α-*Proteobacteria*, respectively [38]. The thermotolerant type I genera *Methylococcus* and *Methylocaldum* can be further classified as type X or type Ib [39]. The phylum *Verrucomicrobiota* exhibits thermophilic and acidophilic characteristics and require rare earth elements to grow and this clade has been designated type III [40, 41]. Methanotrophs in the *Actinomycetota* are only represented by two isolates, “*Ca.* Mycobacterium methanica” and “*Ca*. M. methanotrophicum” [34, 42]. Besides methane, facultative methanotrophs, for instance, *Methylocella* and *Crenothrix* [43–46], are capable of growing on multicarbon substrates, such as short-chain alkanes, acetate, other organic acids, or ethanol [43].

Anaerobic oxidation of methane (AOM) has been found ubiquitously in ecosystems and is driven by ANME archaea and NC10 phylum bacteria. Extant anaerobic methanotrophic archaea (ANME) belong to *Halobacteriota*, previously a division of the *Euryarchaeota* [47]. ANME can be further subdivided into three major groups: (i) ANME-1a/b/c (*Methanophagales*); (ii) ANME-2a/b (*Methanocomedenaceae*), ANME-2c (*Methanogasteraceae*), and ANME-2d (*Methanoperedenaceae*); and (iii) ANME-3 (*Methanosarcinales*) [48]. Of these, ANME-1 and ANME-2 are the most abundant groups, frequently detected in a variety of anoxic habitats, whereas ANME-3 is primarily associated with deep-sea mud volcanoes and marine methane seeps [49–52]. ANME and their associated sulfate-reducing bacteria (SRB) partners can oxidize almost all the methane generated in marine sediments through the reverse methanogenesis pathway [38, 53, 54]. Some ANME can also oxidize methane independently, or in association with metal-reducing bacteria, by utilizing Mn^4+^ [55], Fe^3+^ [56, 57], As^5+^ [58–61], Cr^6+^ [62, 63], anthraquinone-2,6-disulfonate (AQDS), humic acids, or biochar [24, 64] as electron acceptors. Denitrifying anaerobic methane oxidation (DAMO) couples AOM with nitrate or nitrite reduction. ANME-2d archaea (“*Candidatus* Methanoperedens nitroreducens”) utilize nitrate as the terminal electron acceptor, whereas NC10 bacteria use an intra-aerobic pathway in which nitrite is reduced first to nitric oxide and then to dinitrogen and molecular oxygen by the action of nitric oxide dismutase (Nod), enabling oxygen-dependent methane oxidation.

Biomarkers such as 16S rRNA and functional genes have become indispensable tools for exploring the biodiversity of methanotrophs. The functional genes, *pmoA* and *mmoX*, encoding subunits of particulate and soluble methane monooxygenases (pMMO and sMMO), respectively, are the most commonly used molecular markers for assessing aerobic methanotroph diversity [65]. For AOM-related functional gene analysis, *mcrA* (Methyl Coenzyme M Reductase A) is most frequently used and is also the marker gene typically used for methanogen identification and diversity investigation [66, 67].

Despite significant progress in understanding methane cycling in various ecosystems [14, 68, 69], our knowledge of methanotrophic diversity, metabolic adaptation, and activity in cold wetlands remains limited. The physiological and genomic features that enable methanotrophs to oxidize methane efficiently under low temperatures and fluctuating redox conditions are still poorly understood. Therefore, a comprehensive synthesis of current knowledge is required to elucidate how methanotrophs function, adapt, and contribute to methane mitigation in cold wetland ecosystems. In this review, we summarize current knowledge on the ecological distribution, phylogeny, diversity, metabolic mechanisms, and environmental drivers of methanotrophy in cold wetlands. We aim to deepen our understanding of methane oxidation processes in these environments and to provide a scientific basis for developing strategies to mitigate methane emissions from cold ecosystems under the changing climate.

## Subjects and methods

All the references used in the analysis of the distribution patterns of methanotrophs in cold wetlands were collected using the search engines of “Web of Science” and “Google Scholar.” The search terms consisted of two parts: (i) the microbial terms, “methanotrophs,” “psychrotolerant/psychrophilic methanotrophs,” “methane-oxidizing microorganism/microbe,” “aerobic/anaerobic methane-oxidizing bacteria,” and “anaerobic methanotrophic archaea” as well as key genera, *Methylobacter*, *Methylocystis*, *Methylomonas,* NC10, and ANME; (ii) the environmental or habitat-related terms, “cold,” “low temperature,” “Arctic/Antarctica,” “Polar,” “Alpine wetland,” “peatland,” and “permafrost.” Boolean operators (AND/OR) were applied within and between the above two parts to retrieve the maximum number of references. The search period was restricted to 1906 to the present, since the first methanotroph isolate was reported in 1906 [70].

All the references were first checked for their study sites or sampling locations to make sure they met the following standards for cold wetlands: (i) located in cold regions as defined in the US literature [18] (latitude above or below 40°, polar zones, high-altitude plateaus, glacial areas, extensive permafrost, and seasonally snow-covered regions) and (ii) met the standards of the Ramsar definition of wetlands [71]. Studies not meeting these criteria were excluded before the remaining references were assigned to countries according to the sampling locations specified in the publications, without making any geopolitical inferences. Latitudes and longitudes, and altitudes of alpine wetlands were recorded. The wetland types were also confirmed by descriptions of the study sites in each paper. Third, the dominant methanotrophs were identified based on the specific assignment of the taxa and abundance of each taxon. Methanotrophs responsible for aerobic and anaerobic methane oxidation were analyzed separately due to their divergent phylogenetic characteristics.

### Distribution patterns of methanotrophs in cold wetlands

#### Aerobic methanotrophs (MOB) phylogenetic tree analysis and distribution patterns in cold wetlands

**Figure 2:**
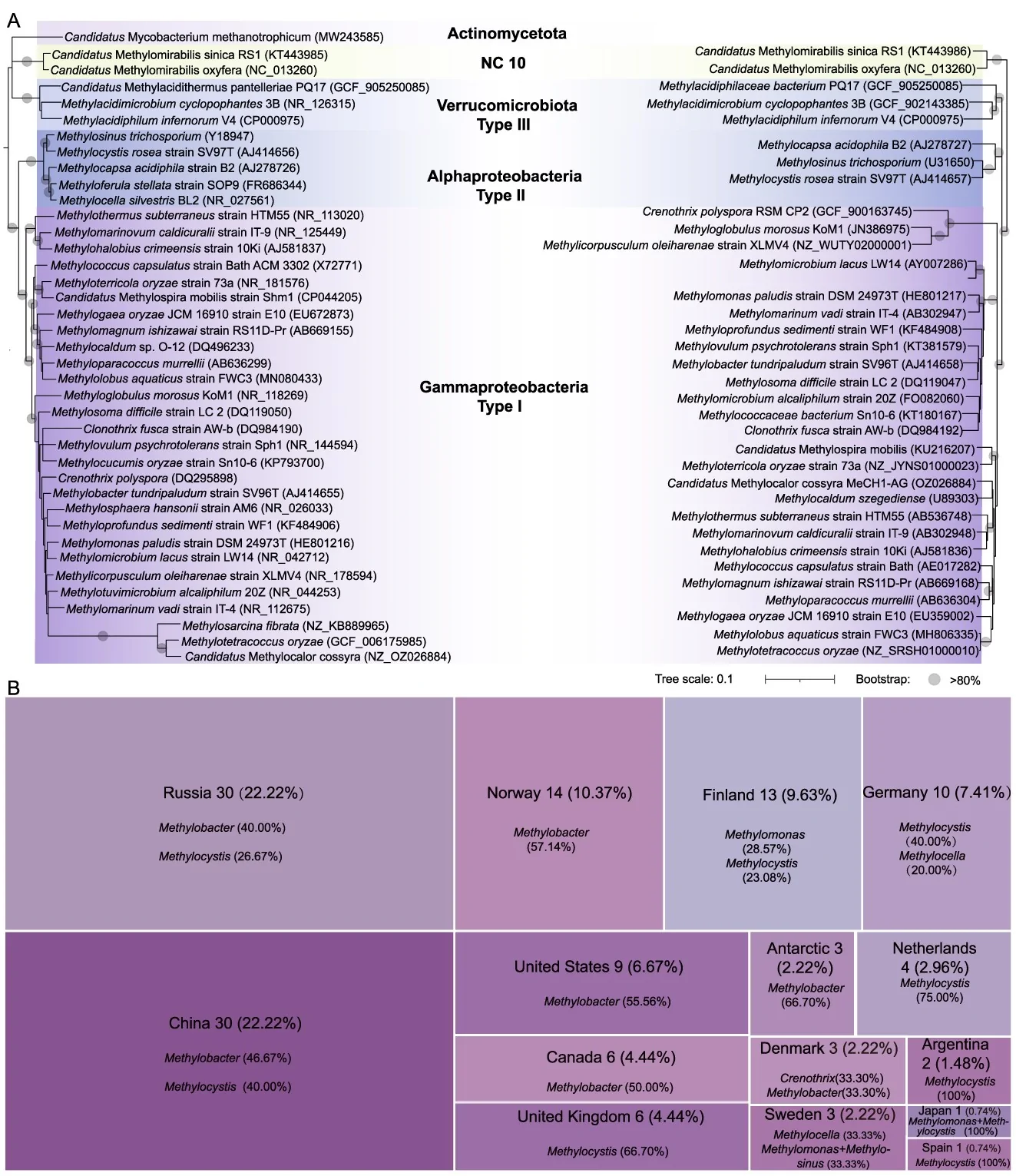
Phylogenetic affiliation of aerobic methanotrophs and geographical distribution of published studies. (A) Phylogenetic tree of bacterial methanotrophs based on 16S rRNA genes (left) and *pmoA* genes (right) using NC10 bacteria as out group. (B) Studies related to aerobic methanotrophs in cold wetlands worldwide. The number of studies in each country and the percentage of the total (in parentheses) are shown. The methanotroph genera most frequently reported as predominant in the reviewed studies from each country are also shown, with the corresponding percentage of studies in parentheses. The criteria for the predominant genera are based on the description of the most abundant methanotrophs in each study. Countries with one representative genus means this group overwhelmingly dominated in most studies (in over 50% of the studies in a country), whereas countries with two representative genera mean the two genera were identified as dominant methanotrophs in most studies (the two genera with a combined frequency of over 50% in over 50% of the studies in a country). Detailed information, including references and statistical analyses, is provided in Table S1.

We collected MOB data from publicly available references and analyzed their phylogenetic attributes. A total of 37 genera of methanotrophs have been identified based on 16S rRNA and *pmoA* genes (Fig. 2A). Except for “*Ca*. Mycobacterium methanica,” the remaining 36 genera were detected by using biomarkers in the cold wetlands (Supplementary Table S1), whereas only eight genera (including *Methylobacter* [72–75], *Methylosphaera* [76], *Methylomonas* [77–79], *Methylocella* [80–83], *Methylocapsa* [84–86], *Methylovulum* [87–90], *Methylocystis* [91–93], and *Methyloferula* [94]) were reported with purified isolates.

For the distribution pattern analysis, a total of 125 MOB-related references were collected worldwide. The characteristics of MOB studies in cold wetlands are shown in Fig. 2B (Table S1). The most frequently investigated cold wetlands were identified from areas in Russia and China (Fig. 2B). Approximately 44.4% (30 cases each) of reported methanotrophic study sites were from these two countries, attributed to the vast areas of cold regions in the two countries [95, 96]. The Qinghai-Tibetan Plateau (QTP) is a hotspot for methanotrophic studies (22 cases) due to its alpine permafrost climate. Other countries, including Finland (14 cases), Norway (13 cases), Germany (10 cases), the United States (9 cases), Canada (6 cases), and the United Kingdom (6 cases), were reported. In contrast, our screening identified only a very limited number of studies (less than 5 cases) from the Netherlands, Sweden, Argentina, Antarctica, and several other countries (Fig. 2B; Table S1).

Based on the summarized studies, type I genus *Methylobacter* and the type II genus *Methylocystis* were the most widespread methanotrophs, occurring individually or together in 14 of the 15 countries examined, reflecting their ecological dominance in cold environments (Fig. 2B; Table S1). Specifically, type I methanotrophs (*Methylobacter* and *Methylomonas*) are responsible for methane oxidation in freshwater wetlands such as marshes, swamps, lakes, as well as saline lakes (60 studies). Type II methanotrophs (*Methylocystis, Methylocella,* and *Methylosinus*) are vital methane consumers in peatlands or peat bogs (58 studies), especially in northern latitudes (Table S1), where peat mosses (*Sphagnum* mosses) flourish and symbiotically attenuate methane with MOB [97].

Besides the common MOB features shared in most cold wetlands, a notable characteristic is that cold environments harbor novel environment-specific methanotrophs. These methanotrophs are phylogenetically distant from their mesophilic relatives, indicating a divergent evolutionary route of cold-adapted methanotrophs on a global scale.

A comprehensive analysis of references in cold wetlands (Tables S1 and S2) revealed that most typical novel methanotrophs are found in high-latitude and polar regions, while the Alpine wetlands of QTP harbor methanotrophic clusters distinct from those of high-latitude regions.

In the northern mountainous wetlands of Norway, several methanotrophs, including *Methylococcus*, uncultured *Methylocaldum*, freshwater cluster LW21, and *Methylocystis/Methylosinus*-affiliated Peat264 lineage, are novel clusters that dominate in the classic Northern moss peatlands [98]. Both LW21 cluster and Peat264 lineage are distantly related to known representatives, and they have also been found in Finland wetland [99], and the Moor House peat bog in the United Kingdom [100], indicating a wide distribution of this clade.

A three-year investigation along an active layer of a permafrost thaw gradient at Stordalen Mire (Abisko, Sweden) discovered thirteen methanotrophic MAGs of which two novel sequences belonged to uncultured upland soil cluster alpha (USCα), with the *mmoX* gene being reported for the first time in this lineage. A potentially methanotrophic *Hyphomicrobiaceae* genome containing methanotrophic marker genes was also recovered, suggesting this should be added to the recognized alphaproteobacterial methanotrophic families [101]. *Methylocapsa* strain D3K7, isolated from northern Russian wetlands, occupies a phylogenetic position between characterized *Methylocapsa* methanotrophs and representatives of the uncultivated USCα lineage, shedding light on these enigmatic but environmentally important Methanotrophs [85].

In high-Arctic Axel Heiberg Island, Canada, metagenomic analysis recovered a novel *Chloroflexi* MAG which contains *mmoX* and harbors genes for copper import, biopolymer synthesis, mercury detoxification, and ammonia uptake [102]. This finding needs further cultivation-based evidence to prove the methane oxidation potential of this novel *Chloroflexi* species. In 2023, from Antarctic freshwater lake sediments, a novel type I methanotroph, “*Candidatus* Methylobacter titanis” closely related to known species *Methylobacter svalbardensis* [75], *Methylobacter tundripaludum* and *Methylobacter psychrophilus*, was obtained using a metagenomic assembly method [103, 104]. The genome of “*Ca.* Methylobacter titanis” harbors genes encoding nitrate reductase *narGHJI* and nitrite reductase *nirBD*, implying they may utilize nitrate/nitrite as nitrogen sources [103]. A dominant cluster of *Methylobacter-*affiliated (uncultured *Methylobacter* clusters I and II) sequences, previously found in an active permafrost layer from the Lena Delta, Siberia in 2008 using the clone library method, is also related to *M. tundripaludum* and *M. psychrophilus [280]*. However, the relationship between these two novel *Methylobacter* species is still unknown.

Alpine wetlands of the QTP represent a typical high-altitude cold wetland. Two novel type I methanotroph MAGs were recovered from the Zoige wetland on the QTP. Both MAGs are distantly related to extant *Methylobacter* and *Methylovulum* at the genome level [105] and contain key genes for methane oxidation, including *pmoCAB* and both *mxaF* and *xoxF* types of methanol dehydrogenase, together with genes for carbon assimilation via the H_4_MPT and RuMP pathways [105]. Additionally, the novel *Methylobacter* was also identified as the dominant methanotroph at the study site [105, 106]. Novel *pmoA* and *mmoX* gene clusters belonging to type I methanotrophs were also detected in the Hongyuan, Riganqiao Nature Reserve on the QTP [107]. A novel *Methylomonadaceae* lineage belonging to type I methanotrophs was also discovered in glacier foreland wetlands on the QTP [108].

Besides being distinct at the genetic level, methanotrophs in cold wetlands also demonstrate different methane oxidation capacities, although clearly care must be exercised in comparing data between different studies. Methane oxidation rate in *Sphagnum* moss peatlands can reach 10.6 μmol CH₄ g^−1^ dw day^−1^ at 15°C [98], and ranged from 0.15 to 6.28 μmol CH₄ g^−1^ dw d^−1^ at 10°C in soils along the thaw gradient of Stordalen Mire [109]. Lake water from Antarctic containing “*Ca.* Methylobacter titanis” maintained methane oxidation rates from 0.02 to 12.9 μmol g^−1^ dw d^−1^ dependent on temperature (5°C–30°C) [103]. A much higher methane oxidation rate was observed in the Zoige wetland of QTP soil (26.7 μmol g^−1^ day^−1^ at 25°C and 15.5 μmol g^−1^ day^−1^ at 10°C, wet weight) [105]. However, only 50 nmol CH₄ g^−1^ dw day^−1^ at the optimal 21°C was measured in Lena Delta, Siberia [20].

#### AOM archaea phylogenetic analysis and AOM methanotrophs distribution patterns in cold wetlands

**Figure 3:**
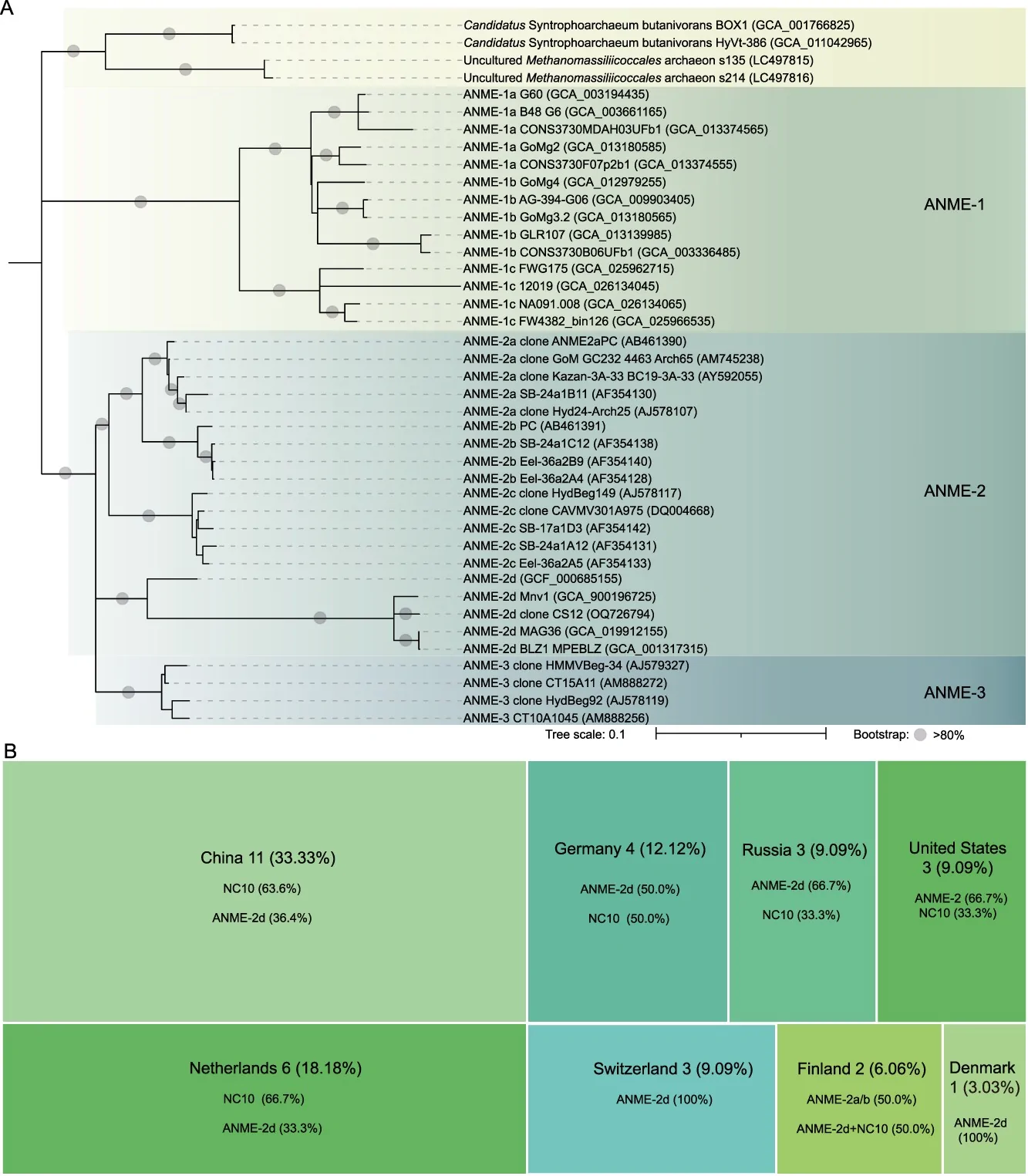
Phylogenetic affiliation of anaerobic methanotrophs and the geographical distribution of published studies. (A) Phylogenetic tree of the ANME archaeal clades based on 16S rRNA genes. (B) Studies related to AOM in cold wetlands worldwide. The number of studies in each country and the percentage of the total (in parentheses) are shown. The anaerobic methanotroph clades most frequently reported as predominant in the reviewed studies from each country are also shown, with the corresponding percentage of studies in parentheses. The criteria for the predominant anaerobic methanotroph clades are based on the description of the most abundant taxa in each study. Countries with two representative clades means both of the two groups were detected with high relative abundances (with a combined percentage equal to 100%). Detailed information, including references and statistical analyses, is provided in Table S2.

Phylogenetic analysis of AOM archaea based on 16S rRNA genes is shown in Fig. 3A. Unlike MOB, all three sub-types of ANME have been detected in cold regions (Supplementary Table S2).

Compared to MOB, studies focusing on AOM in cold wetlands remain relatively limited, and 33 studies were collected. The distribution patterns were also categorized by country (Fig. 3A). Most AOM related studies were carried out in Northern hemisphere, including high latitude and Alpine wetlands. China was found to have eleven publications, most of which were on the QTP (six studies), three in Yunnan Province, and two in Northeast China. In addition, studies from the Netherlands (six studies), Germany (four studies), United States (three studies), Switzerland (three studies), and Russia (three studies) also contributed notable findings. In contrast, only a few studies (fewer than 3) were reported from Finland and Denmark (Fig. 3B; Table S2).

The common trend of AOM distribution in nearly all the cold wetlands are that NC10 bacteria and ANME-2d dominate, either separately or together (Fig. 3B; Table S2). Specifically, in China, the Zoige wetland, a typical alpine cold wetland, NC10 bacteria was shown to dominate by several studies, while the ANME-2d cluster was also identified as a major AOM player [110–113]. Furthermore, a novel ANME-2d species “*Ca*. Methanoperedens psychrophilus” from the Zoige wetland of the QTP was obtained from metagenomic assembly, and soil sediment from the wetland exhibited a methane oxidation rate of 3.87 μmol d^−1^ at 18°C [110].

In Dutch marshes, the major AOM consortia were NC10 bacteria (four wetlands) and ANME-2 archaea (two wetlands) [114–119]. Again, in German freshwater sediments, the predominant anaerobic methanotrophs were NC10 bacteria [120, 121], whereas in marsh environments, ANME-2d archaea were the dominant AOM group [122]. Russian permafrost wetland harbored both NC10 bacteria and ANME-2d archaea, separately, and littoral wetland harbored ANME-2d archaea [123–125]. In Michigan peatlands, USA, the NC10 bacteria were predominant [126], and in permafrost lakes, ANME-2d and ANME-2 archaea were key AOM players. In Arctic permafrost, the main anaerobic methanotrophs detected were ANME-2d archaea [126, 127]. In Finnish estuarine wetland, ANME-2a/b were dominant [128], and both ANME-2d and NC10 bacteria were dominant in lake sediments [129]. In alpine lake sediments from Switzerland, ANME-2d was the only anaerobic methanotroph that dominated in the cold wetlands reported [130–132].

Novel methanotrophic strains of both MOB and AOM have been consistently reported from cold wetlands globally, indicating multiple evolutionary routes in response to these different environments.

### Cold-adapted methanotrophs and their evolution

Why do these cold-adapted methanotrophs from contrasting cold environments function alike but diverge genetically from each other, and why do they exhibit different methane oxidation capacities? These inquiries have been discussed in a publication in 2023, provoking speculation on the evolution of microbial groups in these environments [133]. Explaining the significantly different methane oxidation ability of the methanotrophs in different cold regions, with differing geography, hydrology, and even nutrient availability, will need evidence from “multi-omics” technologies, including genomics, transcriptomics, proteomics, and epigenetics. These holistic studies are essential to predict if these geographically divergent methanotrophs will be capable of removing methane in changing environments.

### Methane oxidation pathways in methanotrophs

**Figure 4:**
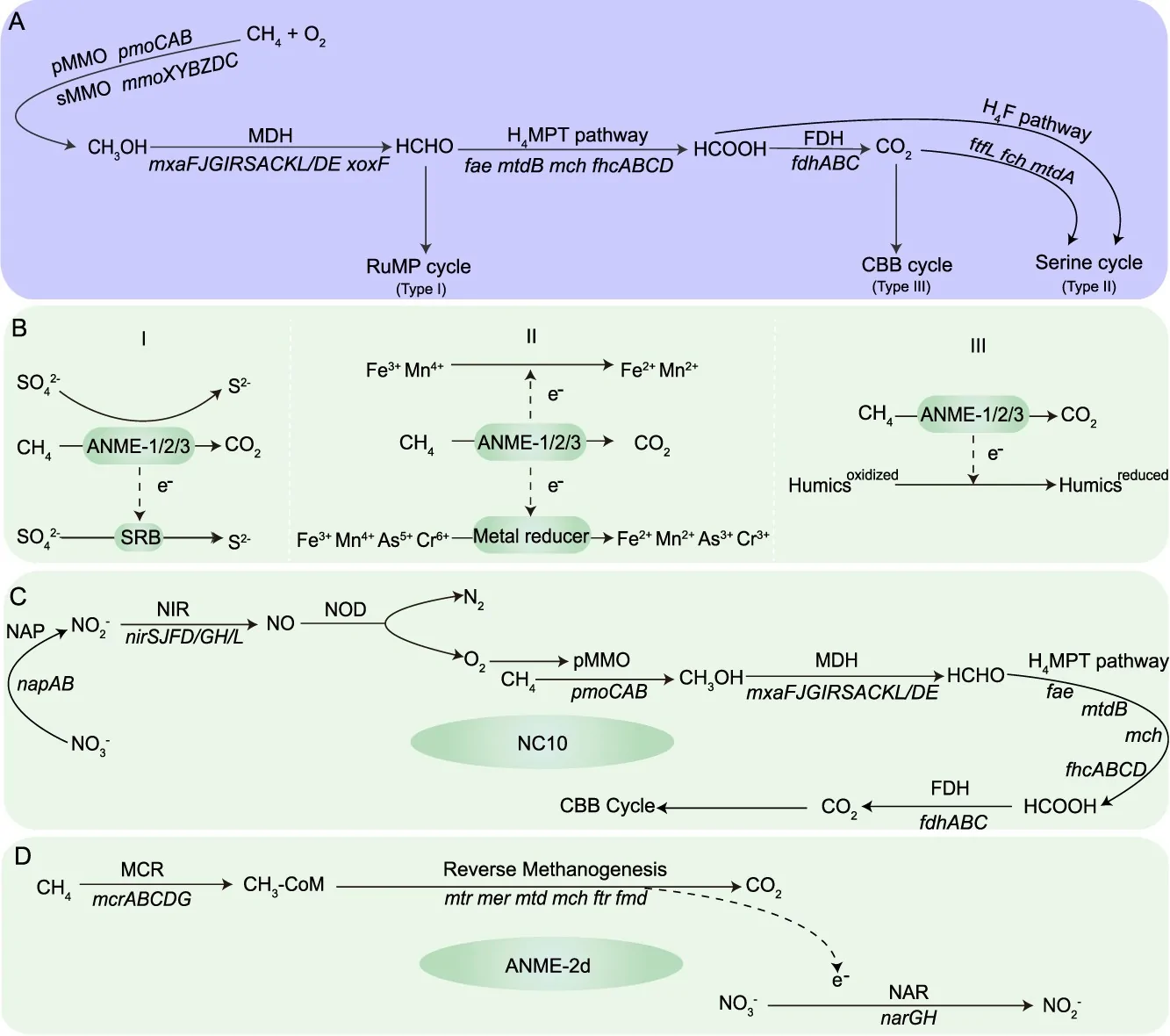
Methane oxidation pathways of methanotrophs [30, 116, 144–147]. (A) Methane oxidation pathways in aerobic methanotrophs [30, 147]. (B) S-dependent AOM pathway mediated by ANME archaea and SRB (I) and metal-AOM pathway mediated by ANME archaea and metal-reducing bacteria (II), ANME archaea-mediated anaerobic methane oxidation pathway with humic substances as electron acceptors (III). (C) Metabolic pathway of nitrate- and nitrite-dependent DAMO mediated by NC10 bacteria*. napAB*, periplasmic nitrate reductase; *nirSJFD/GH/L*, nitrite reductase; *nod*, nitric oxide dismutase; *pmoCAB*, particulate methane monooxygenase; *mxaFJGIRSACKL/DE*, methanol dehydrogenase; H_4_MPT pathway: The associated genes are the same as Fig. 4A; *fdhABC*, formate dehydrogenase; CBB-cycle, Calvin–Benson–Bassham cycle. (D) Nitrate-dependent DAMO pathway mediated by archaeon ANME-2d. *mcrABCDG*, methyl coenzyme M reductase; reverse methanogenesis pathway: *mtr*, N5-methyl-H_4_MPT: CoM methyltransferase; *mer*, N^5^,N^10^-methylene-H_4_MPT reductase; *mtd*, F_420_H_2_-dependent methylene-H_4_MPT dehydrogenase; *mch*, N^5^,N^10^-methenyl-H_4_MPT cyclohydrolase; *ftr*, Formylmethanofuran: tetrahydromethanopterin formyltransferase; *fmd*, Formylmethanofuran dehydrogenase; *narGH*, nitrate reductase [144].

In MOB (Fig. 4A), methanotrophs oxidize methane to methanol utilizing molecular oxygen, catalyzed by either pMMO or sMMO [134–136]. Methanol is subsequently oxidized to formaldehyde by methanol dehydrogenase (MDH) [137, 138]. Type I methanotrophs assimilate carbon at the formaldehyde stage via the ribulose monophosphate (RuMP) pathway. In type II methanotrophs, carbon enters the serine pathway to produce biomass through methylene tetrahydrofolate (H₄F). In this process, formaldehyde is oxidized to formate via methylene tetrahydromethanopterin (H₄MPT [139–142] (Fig. 4A). Type III methanotrophs fix carbon dioxide via the Calvin–Benson–Bassham (CBB) cycle [143].

In AOM processes (Fig. 4B-D), the reverse methanogenesis pathway is coupled with the reduction of various electron acceptors including SO₄^2−^, Fe^3+^, Mn^4+^, Cr^6+^, As^5+^, humic acids, or biochar (Fig. 4B).

DAMO processes have a significant role in methane oxidation in freshwater marshes and wetlands with low SO_4_^2−^ concentrations [148]. In the first-described NC10-mediated DAMO, nitrite is initially reduced to nitric oxide (NO) by nitrite reductase. The generated NO is further converted into nitrogen and oxygen by an as-yet uncharacterized nitric oxide dismutase (Nod). The NC10 bacteria possesses all the genes necessary for conventional aerobic methane oxidation (encoding pMMO and MDH) (Fig. 4C). Alternatively, nitrate-dependent archaeal DAMO also recruits the reverse methanogenesis pathway for methane oxidation. The ANME-2d archaeon generates electrons via the reverse methanogenesis pathway. Nitrate is reduced to nitrite by nitrate reductase (NAR) (Fig. 4D).

### Cold-adapted mechanisms in psychrotolerant/psychrophilic methanotrophs

Psychrophilic microorganisms survive and reproduce in cold environments through a series of complex mechanisms, involving adjustments to cell structure, metabolic activities, and molecular functions [149]. Cellular strategies like cell membrane stability, up-regulation of peptidoglycan biosynthesis, increased production of extracellular polymeric substances, and adoption of membrane pigmentation [150]. Metabolic adjustments include downregulation of primary pathways and adoption of alternative or simplified secondary metabolic routes to overcome nutrient uptake limitations in cold environments [27]. Microbial cold adaptions should be explored by using “omics” technologies to explain molecular adaption at genomic, transcriptomic, and proteomic levels [27].

Research on the cold-adaptation mechanisms of methanotrophs in cold wetlands remains limited. A study on Zoige wetland soils of the QTP [110] identified an iron (III) dependent AOM, identified as the novel ANME species “*Ca.* Methanoperedens psychrophilus.” Comparative genomic analyses revealed that “*Ca. M. psychrophilus*” contained unique gene categories, including a multi-heme cluster (MHC) cytochrome, S-layer protein-coding genes homologous to others from cold environments, and type IV pili coding gene. These features likely confer cold adaptability in “*Ca. M. psychrophilus*,” enabling it to participate in AOM under low-temperature conditions. The low temperature of the Zoige wetlands might have triggered cold-adapted mechanisms in methanotrophs, enhancing their survival in the extreme environment.

Cold-adaptive mechanisms in methanotrophs have never been illustrated clearly. However, genomic analysis in several psychrophilic/psychrotolerant methanotrophs has been published to explain their metabolic characteristics. Four isolates, *M. svalbardensis* LS7-T4A^T^ [75], *Methylobacter* sp. S3L5C [72] from Arctic, *Methylovulum psychrotolerans* S1L and *Methylomonas paludis* S2AM from northern lake water [87], share common traits of type I methanotrophs, but each also has its own traits. The transport system of *M. svalbardensis* LS7-T4A^T^ comprises periplasmic substrate-binding proteins (*pstS*), membrane-associated components (*pstA* and *pstC*), and cytoplasmic phosphate-release proteins (*pstB*), indicating efficient nutrient acquisition under cold conditions [75]; *Methylobacter* sp. S3L5C carries genes encoding key enzymes for organic acid production (*ackA*, *acdAB*, *mdh*), enabling methane conversion to organic acids, which can provide nutrients to other microorganisms in the environment. MOB strains obtained from Finnish lakes, including *Methylovulum psychrotolerans* S1L and *Methylomonas paludis* S2AM, also harbor genes predicted to drive organic acid formation [72, 87]. Such organic acid production serves as an energy-saving strategy that likely sustains microbial growth and metabolism under low-temperature conditions. Siberian psychrotolerant strain *Methylovulum psychrotolerans* Sph1^T^ contains the complete set of glycogen biosynthesis genes (*glgA*, *glgB*, *glgC*) and shows potential for hopanoid synthesis, which is essential for cytoplasmic membrane maintenance [89].

These features highlight the metabolic basis of cold-adaptative MOB. Further studies focusing on the unique traits of psychrophilic/psychrotolerant methanotrophs at transcriptomics and proteomics levels are necessary to untangle the complex issues and to decide what are the key strategies that control the growth and methane oxidation potential of methanotrophs under cold stress.

### The methane oxidation mechanism of aerobic methanotrophs under microaerophilic/anoxic conditions

Methane oxidation can also occur under microaerophilic or anoxic conditions. Research into the distribution and metabolism of aerobic methanotrophs in oxygen-deprived aquatic environments identified four primary metabolic strategies under anoxic or low-oxygen conditions: (i) oxygen production or storage by the methanotrophic bacteria, (ii) consortium formation with oxygenic microorganisms, (iii) forming associations with non-oxygenic heterotrophic bacteria that utilize alternative electron acceptors, and (iv) utilization of alternative electron acceptors instead of oxygen [151, 152].

Genomic analysis of the micro-aerobic methanotroph “*Ca.* Methylospira mobilis” Shm1, isolated from northern freshwater wetlands, revealed that its genome encodes three copies of bd-type terminal oxidases with high oxygen affinity, enabling efficient respiration under low-oxygen conditions. Its genome also contains a full set of motility genes, facilitating rapid localization of optimal oxygen and substrate concentration zones in microaerobic environments, through which it can sustain growth, metabolic activity, and methane oxidation [153]. Moreover, a study of the water column in Lake Zug, Switzerland, found that methanotrophs from the *Methylococcales* exhibited methane assimilation under both anoxic and aerobic conditions. Genomic and transcriptomic analyses revealed that *Methylococcales* harbored the complete *pmoCAB* gene cluster. Under anoxic conditions, most genomes exhibited upregulation of genes associated with fermentation and denitrification, suggesting that aerobic methanotrophs may shift toward fermentation-based methane metabolism coupled with denitrification. Hence, the metabolic versatility of *Methylococcales* in such hypoxic environments likely enhances their survival [154].

Using other substrates as electron acceptors is an alternative for methanotrophs under hypoxic conditions. Studies of lake sediments from the Northern Brooks Range in Alaska found that *Methylobacter-*associated type I methanotrophs dominated the site. Methane oxidation here likely occurs in association with iron reduction under oxygen-limited conditions at the lakebed. Metagenomic analysis revealed genes associated with nitrogen cycling and hydrogen metabolism, which was hypothesized to be part of an energy-saving strategy employed by *Methylobacter* in response to hypoxic conditions [155]. DNA-SIP and PLFA-SIP based studies further indicate that *Methylobacter* may utilize iron or manganese as electron acceptors for methane oxidation under anoxic conditions [156]. A metagenomic study of a high-altitude, iron-rich freshwater lake in Yunnan province found that among the six high-quality MAGs, genes related to riboflavin metabolism, extracellular electron transfer (EET), conductive pili (*pilA*), and MHCs, were detected. In the absence of oxygen, methanotrophs utilize electron shuttling molecules like riboflavin and perform EET, using iron oxides (particularly Fe(III) oxides) as alternative electron acceptors to facilitate methane oxidation [151].

### The impact of environmental factors on methane oxidation activity and regulatory mechanisms in cold wetlands

Current evidence indicates that temperature, hydrology, oxygen availability, substrate supply, nutrient status, and microbial interactions are key factors in shaping methane oxidation and methanotroph diversity in cold wetlands. Temperature influences methane oxidation capacity both directly, and indirectly by changing the microbial community composition [20, 157]. In Arctic lake sediments and Siberian permafrost, type I methanotrophs are dominant, whereas warming alters the dominant taxa and increases the ecological importance of type II methanotrophs [157, 158]. Meanwhile, some psychrotolerant strains (e.g. *M. tundripaludum* SV96) can maintain methane-oxidizing activity at low temperatures through physiological acclimation, a process further modulated by methane availability [159]. Besides temperature, nutrient availability also influences methanotroph community structure. On the QTP, ammonium, nitrate, and urea fertilizers shifted dominance from type II *Methylocystis* to type I *Methylomonas* [160].

Freeze–thaw cycles (FTCs) can regulate methane oxidation by restructuring hydrology, redox conditions, and the active microbial layer [161–163]. FTCs also affect cumulative methane emissions [164]. Along permafrost thaw gradients, methane oxidation is significantly constrained by porewater redox conditions and water-table position, with highest activity under low redox potential and high water tables [109]. Furthermore, freeze–thaw also disrupts microbial physiology via ice-crystal formation and osmotic stress, affecting membrane stability, dehydration-rehydration responses, and cell lysis-driven substrate and nutrient release, thereby reshaping microbial metabolism [165]. Although direct evidence for methanotroph-specific responses to freeze–thaw remains rare, thaw has been shown to rapidly reorganize microbial communities, functional genes, and GHG-related metabolism [166, 167].

Notably, viruses and other extrachromosomal genetic elements (ECEs) can also influence methane oxidation by intervening in the host metabolism. A recent study based on metagenomic analysis of almost 1000 diverse host-associated and environmental habitats identified 24 distinct auxiliary metabolic genes (AMGs), predicted to be involved in methane metabolism, which were encoded by 911 viral genomes. These AMGs may influence methane metabolic pathways by modulating the metabolism of their respective hosts upon infection. The extent to which viruses employ these AMGs to alter host metabolism appears to be shaped by the ecological characteristics of their habitats [168]. A large phage has been reported to enhance methane oxidation in a freshwater lake. The *pmoC* sequence of *pmoC-*phage shares >90% identity with those of coexisting methanotrophs (e.g. *Methyloparacoccus*, *Methylocystis,* and *Methylobacter*), suggesting horizontal gene transfer between the methanotrophs and their associated phages. CRISPR-Cas analyses predicted host-phage interactions, and transcriptomic data showed high *pmoC* expression in phages, supporting their involvement in upgrading host methane oxidation rates [169]. In addition to viruses, 23 novel ECEs, termed “mini-Borgs,” have been identified from the ANME archaeon *Methanoperedens* in wetland soils. Evidence suggests horizontal gene transfer between mini-Borgs and the host, potentially influencing physiology and evolution of *Methanoperedens*, which in turn affects the methane cycle [170].

### Perspectives

Methanotrophs in cold wetlands play an indispensable role in mitigating methane emissions to the atmosphere. Both MOB and AOM methanotrophs showed unique distribution patterns across the entire cold wetlands and developed novel and unique methanotrophic clusters during long time-periods of evolution. Current evidence suggests that psychrophilic and psychrotolerant methanotrophs adapt to cold by maintaining core metabolism and enhancing resource acquisition ability and metabolic flexibility. Future research should focus on both theoretical and applied aspects of methanotrophy to enhance our understanding of the ecological functions and to harness the potential for GHG mitigation. The following directions are particularly promising:

(1) Elucidating the metabolic and regulatory mechanisms of novel cold-adaptive methanotrophs, and their global distributions. This can be achieved by a combination of metagenomics, single cell genomics, transcriptomics, proteomics, and metabolomics. Studies at the levels of genetic potential, gene expression, protein expression, and substrate cycling will help to explain the in situ activities of methanotrophs.
(2) Isolation of cold-adapted/psychrophilic methanotrophic strains and exploration of their bio-physicochemical characteristics. Conventional isolation techniques combined with microfluidics-based single cell high-throughput chips will greatly improve isolation efficiency.
(3) Exploring the interactions involved in plant-methanotrophs symbioses in cold wetlands. The interaction between plants and methanotrophs is crucial to methane oxidation in wetlands but this process has long been ignored. Studies in this direction will clarify the importance of plants to methane oxidation in cold wetlands.
(4) Decoding the key enzymes involved in cold-adaptive/psychro-philic methane oxidation processes by recruiting artificial intelligence tools, leading to enhanced expression and activity of key enzymes by using gene editing technologies.

## Supplementary Material

R4_Supplementary_Information_ATC_ycag214

## Data Availability

Data sharing is not applicable to this article as no datasets were generated or analyzed during the current study.
