# Supplementary material for: Methanotrophs and methane oxidation in cold wetlands: distribution, ecology, and mechanisms
Source: ISME Commun. 2026 Jul 26;6(1):ycag214. doi: 10.1093/ismeco/ycag214 (PMC13518059; doi:10.1093/ismeco/ycag214)
Supplement: Supplementary file 1 [file r4_supplementary_information_atc_ycag214.docx]

**Supplementary Information**

**Methanotrophs and methane oxidation in cold wetlands: distribution, ecology and mechanisms**

Lerui Zhou^1^, Juanli Yun^1*^, Yongcui Deng^2^, Kai Xue^3^, Muhammad Farhan Ul Haque^4^，Andrew Crombie^5^, Anzhou Ma^3,6*^, Yanfen Wang^3*^

^1^ College of Environmental Science and Technology, Shaanxi University of Science and Technology, Xi’an 710021, China

^2^ School of Geography, Nanjing Normal University, Nanjing 210023, China

^3^ College of Resources and Environment, University of Chinese Academy of Sciences, Beijing 100049, China

^4^ School of Biological Sciences, University of the Punjab, Quaid-i-Azam Campus, Lahore 54590, Pakistan

^5^ School of Biological Sciences, University of East Anglia, Norwich NR4 7TJ, United Kingdom

^6^ Research Center for Eco-Environmental Sciences, Chinese Academy of Sciences, Beijing 100085, China

**Running title:** Methanotrophs in cold wetlands.

***Corresponding authors:**

Juanli Yun: College of Environmental Science and Technology, Shaanxi University of Science and Technology, Xi’an 710021, China. E-mail: [yunjl@sust.edu.cn;](mailto:yunjl@sust.edu.cn;)

Anzhou Ma: Research Center for Eco-Environmental Sciences, Chinese Academy of Sciences, Beijing 100085, China; College of Resources and Environment, University of Chinese Academy of Sciences, Beijing 100049, China. E-mail: azma@rcees.ac.cn；

Yanfen Wang: College of Resources and Environment, University of Chinese Academy of Sciences, Beijing 100049, China. E-mail: yfwang@ucas.ac.cn

**The supplementary information includes the following:**

Tables S1-S2

References

**Supplementary Tables**

**Table S1** Reference list of MOB studies in this review, the predominant methanotroph genera reported in each study, and the most frequently reported predominant genus in the reviewed studies from each country.

| **Country^*^** | **Study site** | **Location** | **Wetland type** | **Dominant methanotrophs** | **Ref.** | **Number of studies in each country** | **Percentage of studies that dominant MOB were detected in each country** |
| --- | --- | --- | --- | --- | --- | --- | --- |
| **Antarctica** | Fildes Peninsula in the southwest of King George Island | 62°08′S-62°14′S, 59°02′W-58°51′W | Freshwater Lake | *Methylobacter* | ^[1]^ | 3 | *Methylobacter* (2/3)=66.70% |
|  | Fildes Peninsula in the southwest of King George Island | 62°08′S-62°14′S, 59°02′W-58°51′W | Freshwater Lake | *Methylobacter* | ^[2]^ |  |  |
|  | Eastern Antarctica | 68°S ,78°E | Salinity Lake | *[Methylosphaera](https://www.microbiologyresearch.org/content/journal/micro/10.1099/00221287-143-4-1451)* | ^[3]^ |  |  |
| **Argentina** | Tierra del Fuego | 54°48′S, 68°18′W | Mosses from Pool, Lawn and Hummock | *Methylocystis* | ^[4]^ | 2 | *Methylocystis* (2/2)=100% |
|  | Tierra del Fuego | 54°45′S, 68°20′W;  54°58′S, 66°44′W | Peat Bog | *Methylocystis* | ^[5]^ |  |  |
| **Canada** | Axel Heiberg Island | 79°26'N, 90°46′W | Tundra | *Mmethylocapsa*, *Methylmarinovum* | ^[6]^ | 6 | *Methylobacter* (3/6)=50.00% |
|  | Herschel Island | 69°34′N, 138°57′W | Tundra | *Methylocystis* | ^[7]^ |  |  |
|  | Northern Quebec | 58.06°N, 68.24°W | Tundra | *Methylosinus, Methylocystis* | ^[8]^ |  |  |
|  | Yukon Territory | 62.16°N-62.61°N; 140.68°W-140.99°W | River | *Methylobacter* | ^[9]^ |  |  |
|  | Herschel Island ;Beaufort Coast | 69°35′N, 139°00′W;  69°02′N, 133°55′W | Permafrost | *Methylobacter* | ^[10]^**^c^** |  |  |
|  | Subarctic Québec, Four subarctic valleys | 55°20′N, 77°30′W  55°13′N, 77°42′W, 56°37′N, 76°13′W  56°55′N, 76°22′W | Freshwater Pond | *Methylobacter* | ^[11]^ |  |  |
| **China** | Qinghai Lake  3212−3221 m;  3194−3226 m;  3720−3850 m | 36°42′N, 100°46′E;  37°04′N, 99°54′E;  37°43′N,100°08′E | Marshes/Lakeside/Headwater | *Methylococcus* | ^[12]^ | 30 | *Methylobacter* (14/30)=46.67%  *Methylocystis* (12/30)=40.00% |
|  | Namco on the Tibetan Plateau 4725 m;  Sanjiang Plain | 30°46′N, 90°59′ E;  47°35′N, 133°31′E | Freshwater Lake/Marsh | *Methylobacter* | ^[13]^ |  |  |
|  | Sanjiang Plain,  Haibei Station of Tibet Plateau, 3225 m | 47°35′N, 133°38′E;  37°33′N, 101°21′E | Freshwater/Alkaline Marsh | *Methylobacter* | ^[14]^ |  |  |
|  | Xianghai | 44°55′N-45°09′N, 122°05′N-122°31′E | Freshwater Marsh | *Methylobacter* | ^[15]^ |  |  |
|  | Xianghai, Zhalong | 47°35′N, 133°31′E | Freshwater Marsh | *Methylobacter* | ^[16]^ |  |  |
|  | Songneng Plain | 44°N, 124°E | Freshwater Marsh | *Methylocystis* | ^[17]^ |  |  |
|  | Sanjiang Plain | 47°35′N, 133°31′E | Freshwater Marsh | *Methylocystis*, *Methylobacter* | ^[18]^ |  |  |
|  | Sanjiang Plain | 48°3′N, 134°32′E | Freshwater Marsh | *Methylococcaceae* | ^[19]^ |  |  |
|  | Yunnan Plateau,  1273-2555 m | 23.43°N-28.68°N, 99.28°E-103.98°E | Freshwater Lake | *Methylobacter*, *Methylosoma* | ^[20]^ |  |  |
|  | Zoige Reserve, 3445 m | 33°56′N, 102°52′E | Freshwater Lake | *Methylobacter* | ^[21]^ |  |  |
|  | Zoige Reserve,  3430-3460 m | 33°56′N, 102°52′E | Freshwater Marsh | *Methylobacter* | ^[22]^ |  |  |
|  | Tibetan Plateau Lakes,  3000-5000 m | 28.50°N-35.50°N,  79.00°E-100.50°E | Freshwater /Brackish Lakes/Saline lakes | *Methylobacter* | ^[23]^ |  |  |
|  | Lakes Located on the Tibetan Plateau, 4000 m  Zongxiong Co, Qige Co,  Bangong Co ,Long Co,  Zhangnai Co ,Lagor Co | 33°06′N, 80°09′E；  31°11′N, 85°31′E；  33°31′N, 79°50′E；  29°12′N, 87°24′E；  31°32′N, 87°23′E；  32°03′N, 84°10′E； | Freshwater and Saline Lakes | *Methylobacter* | ^[24]^ |  |  |
|  | Yang Lake, 4440 m;  Qinghai Lake, 3195 m | 29.03°N,90.42°E;  36.94°N,99.61°E | Freshwater and Saline Lakes | *Methylovulum* | ^[25]^ |  |  |
|  | Shaliu River, 3197-3199 m  Heima River, 3197-3201 m  Daotang River,3201-3203 m | 37°12′N, 100°11′E;  36°43′ N, 99°47′ E;  36°34′ N, 100°44′E | Estuarine and Nearshore | *Methylobacter*,  *Methylosinus*, *Methylocystis* | ^[26]^ |  |  |
|  | Riganqiao Reserve,  3400 m | 33°06′N, 102°38′E | Peatland | *Methylocystis* | ^[27]^ |  |  |
|  | Hongyuan-Wetland, 3459 m ;  Dangxiong-Wetland, 4290 m;  Maduo-Wetland, 4229 m | 33°06′N, 102°38′N,  30°20′E, 91°03′N; 34°38′E, 98°01′E | Peatland | *Methylocystis* | ^[28]^ |  |  |
|  | Hongyuan-Wetland, 3459 m ;  Dangxiong-Wetland, 4290 m;  Maduo-Wetland, 4229 m | 33°06′N, 102°38′N,  30°20′E, 91°03′N; 34°38′E, 98°01′E | Peatland | *Methylocystis* | ^[29]^ |  |  |
|  | Greater Khingan Mountains | 52°56′N, 122°51′E | Peatland | *Methylocystis* | ^[30]^ |  |  |
|  | Dajiuhu peatland, 1730 m | 31°24′N-31°33′N, 109°56′E-110°11′E | Peatland | *Methylosinus* | ^[31]^ |  |  |
|  | Zoige Reserve, 3445 m | 33°56′N, 102°52′E | Marsh | *Methylocystis* | ^[32]^ |  |  |
|  | Zoige Reserve, 3445 m | 33°56′N, 102°52′E | Marsh | *Methylobacter* | ^[33]^ |  |  |
|  | Zoige Reserve, 3445 m | 33°56′N, 102°52′E | Marsh | *Methylocystis* | ^[34]^ |  |  |
|  | Mitika Alpine Wetland, Northern Xizang  4918 m | 30°51′N-31°09′N, 92°45′E-93°19′E | Marsh-Meadow | *Methylocystis*, *Methylococcus*, *Methylocapsa* | ^[35]^ |  |  |
|  | Yang Lake, 4438 m;  Qinghai Lake/ Meadow,3117 m;  Qinghai Lake,3116 m  Qiangyong Glacier Meadow,4854 m | 29.04°N, 90.42°E;  36.91°N,99.61°E;  36.95°N, 99.60°E;  28.89°N, 90.23°E; | Lake,  Meadow,  Glacier | *Methylocystis*, *Methylobacter* | ^[36]^ |  |  |
|  | Maqu,  3450 m | 33°40′N, 101°52′E | Alpine Swamp | *Methylobacter*, *Methylosinus* | ^[37]^ |  |  |
|  | Longxiazailongba(LXZ) Glacier, 5240-6000 m | 33°5′N-33°6′N, 92°00′E-92°1′E | Swamp Meadow | *Methylobacter* | ^[38]^ |  |  |
|  | Tibetan  Glacier  Foreland, 6000 m | 33°5′N, 92°05′E | Swamp Meadow | *Methylocystis* | ^[39]^ |  |  |
|  | Tibetan Glacier Foreland,  6000 m, 5600 m | 33°5′N, 92°05′E;  36.94°N, 99.61°E | Glacier Foreland | *Methylocystis* | ^[40]^ |  |  |
|  | Proglacial lakes, Western Nyainqentanglha Mountain  5500 m | 29.864°N, 90.203°E | Glacier Lake | *Methylobacter* | ^[41]^ |  |  |
| **Denmark** | Flakkerhuk of West Greenland | 69°40′N, 52°00′W | Permafrost Fen | *Crenothrix* | ^[42]^ | 3 | *Crenothrix* (1/3)=33.30%  *Methylobacter* (1/3)=33.30% |
|  | Greenland | 69°15′N, 53°34′W | Permafrost | *Methylobacter* | ^[10]^**^c^** |  |  |
|  | Denmark of Wetland | 54.5°N-57.8°N, 8.1°E-13°E | Bog, Mire and Fen | *Methylocystis/*Methylosinus | ^[43]^ |  |  |
| **Finland** | Littoral Wetland of Lake Kevätön | 63°6′N, 27°37′E | Littoral | *Methylocystis*, *Methylocysinus* | ^[44]^ | 13 | *Methylocystis* (3/13)=23.08%; *Methylomonas* (4/13)=28.57% |
|  | Littoral Wetland of Lake Kevätön | 63°6′N, 27°37′E | Littoral | *Methylocystis*, *Methylocysinus* | ^[45]^ |  |  |
|  | Jyvaskyla | 62°11′N, 25°40′E | Forest peatland | *Methylocystis* | ^[46]^**^b^** |  |  |
|  | Lakkasuo | 61°48′N, 24°19′E | Fen/Bog/Peatland | *Methylococcus* | ^[47]^ |  |  |
|  | Boreal Forest Soil Inland | 62°11′N, 25°40′E | Peatland | *Methylocella* | ^[48]^**^c^** |  |  |
|  | Siikajoki,Gulf of Bothnia Coast | 64°45′N, 24°42′E | Peatland | *Methylomonas* | ^[49]^ |  |  |
|  | Lake Kevätön and Lake Pöljä | 62°31′N, 29°22′E  62°02′N, 29°59′E | Lake | *Methylococcales* | ^[50]^ |  |  |
|  | Various Boreal Lakes, Southern Finland | 61°00′N, 24°E-25°E | Lake | *Methylomonas*, *Methylovulum* | ^[51]^ |  |  |
|  | Lake Alinen Mustajärvi, Lake Lovojärvi | 61°13′N, 25°8′E;  61°04′N, 25°02′E | Lake | *Methylococcales* | ^[52]^ |  |  |
|  | Lake Lovojarvi and Lake Ormajarvi | 61°04′N, 25°02′E;  61°12′N, 25°06′E | Lake | *Methylomonas*, *Methylovulum* | ^[53]^ |  |  |
|  | Lake Mekkojärvi and Lake Alinen Mustajärvi | 61°13′N, 25°8′E;  61°12′N, 25°06′E | Lake | *Methylobacter* | ^[54]^ |  |  |
|  | Lake Lovojärvi | 61°04'N, 25°02'E | Lake | *Methylobacter* | ^[55]^ |  |  |
|  | Lake Alinen Mustajärvi | 61°13′N, 25°8′E | Lake | *Methylomonas* | ^[56]^ |  |  |
| **Germany** | Teufelssee, Germany | 52°29′N, 13°14′E | Peat bog | *Methylocystis* | ^[57]^ | 10 | *Methylocystis* (4/10)=40.00%；  *Methylocella* (2/10)=20.00% |
|  | Teufelssee, Germany | 52°29′N, 13°14′E | Peat bog | *Methylocystis* | ^[58]^ |  |  |
|  | Teufelssee, Germany | 52°29′N, 13°14′E | Peat bog | *Methylocystis* | ^[59]^ |  |  |
|  | Heiligensee and Hütelmoor | 54°12′N, 12°10′E | Fen Complex | *Methylococcales* | ^[60]^ |  |  |
|  | Heiligensee and Hütelmoor | 54°12′N, 12°10′E | Fen Complex | *Methylocystaceae* | ^[61]^ |  |  |
|  | Lake Kleine Fuchskuhle | 53°10′N, 13°02′E | Peatland | *Methylocystis* | ^[62]^**^a^** |  |  |
|  | Marburg, Germany | 50°48′N, 8°46′E | Peatland | *[Methylocella](https://www.microbiologyresearch.org/content/journal/ijsem/10.1099/ijs.0.02481-0)* | ^[63]^ |  |  |
|  | Marburg, Germany | 50°49′N, 8°46′E | Peatland | *[Methylocella](https://journals.asm.org/doi/abs/10.1128/jb.00506-10)* | ^[64]^ |  |  |
|  | Elbe River,Hamburg Area | 53°32′N, 9°56′E | River | *Methylomonas* | ^[65]^ |  |  |
|  | Marburg, Germany | 50°49′N, 8°46′E | Brook | *Methylocapsa* | ^[66]^ |  |  |
| **Japan** | Bibai, Hokkaido, | 43°19′N, 141°48′E | Peatland | *Methylocystis*, *Methylomonas* | ^[67]^ | 1 | *Methylomonas+Methylocystis* (1)=100% |
| **Netherlands** | Oude Waal | 51°52′N, 05°53′E | Oxbow Lake | *Methylocystis, Merhylobacter* | ^[68]^ | 4 | *Methylocystis* (3/4)=75.00% |
|  | Mariapeel Nature Reserve, Hatertse | 51°24′N, 5°54′E,  51°47′N, 5°48′E | Peatland | *Methylocystis,* *Methylomonas* | ^[69]^ |  |  |
|  | Mariapeel Nature Reserve, Hatertse Vennen | 51°24′N, 5°54′E, 51°47′N, 5°48′E | Peatland | *Methylocystis*, *Methylosinus* | ^[70]^ |  |  |
|  | Mariapeel Nature Reserve | 51°24′N, 5°54′E | Peat Bog | *Methylocella,*  *Methylocapsa* | ^[71]^ |  |  |
| **Norway** | Island of Svalbard | 78°56′N, 11°53′E | High Arctic | *Methylobacter* | ^[72]^ | 14 | *Methylobacter* (8/14)=57.14% |
|  | Island of Svalbard | 78°56′N, 11°53′E | Arctic | *Methylocystis* | ^[73]^ |  |  |
|  | Island of Svalbard | 78°56′N, 11°53′E | Arctic | *Methylobacter* | ^[74]^ |  |  |
|  | Island of Svalbard | 78°56′N, 11°53′E | High Arctic | *Merhylobacter*, *Methylocysinus* | ^[75]^ |  |  |
|  | Island of Svalbard | 78°11′N, 15°55′E | Arctic | *Methylocella* | ^[48]^**^c^** |  |  |
|  | Lagoon Pingo, Central Spitsbergen, | 78°14′N, 15°45′E | Lagoon Pingo | *Methylobacter* | ^[76]^ |  |  |
|  | Solvatn, Knudsenheia | 78°55′N, 11°56′E  78°56′N, 11°49′E | Peatland | *Methylobacter* | ^[77]^ |  |  |
|  | Northern Norway | 69°41′N, 29°12′E | Peatland | *Methylocapsa* | ^[78]^ |  |  |
|  | Northern Norway | 69°69′N, 29°38′E | Peatland | *Methylocystis* | ^[79]^ |  |  |
|  | Spitsbergen | 78°55′N, 11°56′E | Peatland | *Methylobacter* | ^[80]^ |  |  |
|  | Northern Norway | 69°41′N, 29°11′E | Peatland | *Methylocapsa* | ^[81]^ |  |  |
|  | Solvatn, Knudsenheia ，Norway | 78°55′N, 11°56′E；  78°56′N, 11°49′E | Peatland | *Methylobacter* | ^[82]^ |  |  |
|  | Solvatn | 78°55′N, 11°56′E | Peatland | *Methylobacter* | ^[83]^ |  |  |
|  | Langtjern | 60°22′N, 9°43′E | Floodplain, Marsh, Peatland | *Methylocaldum,* Peat group*,*LW2 group | ^[84]^ |  |  |
| **Russia** | Shore of the River Kosaya, River Dudinka | 69°42′N, 86°53′E | River | *Methylocystis* | ^[85]^ | 30 | *Methylobacter* (12/30)=40.00%; *Methylocystis* (8/30)=26.67% |
|  | Siberia | 67.44°N-67.54°N, 86.59°W-86.71°W | Peatland, Lake and Pond | *Methylocystis, Methylomonas* | ^[86]^**^a^** |  |  |
|  | Siberia | 64°51′N, 147°43′W | Permafrost | *Methylobacter* | ^[10]^**^c^** |  |  |
|  | Yamal-Nenets Autonomous Okrug, NW Siberia | 65°35′N, 073°03′E | Acidic Peat | *Candidatus Methylospira mobilis* | ^[87]^ |  |  |
|  | Lena Delta, Siberia | 72°22′N, 126°28′E | Lena Delta | *Methylobacter*, *Methylosarcina* | ^[88]^ |  |  |
|  | Lena Delta, Siberia | 72°37′N, 126°47′E | Lena Delta | *Methylocystis* | ^[89]^ |  |  |
|  | Lena Delta, Siberia | 72°22′N, 126°28′E | Lena Delta | *Methylobacter* | ^[90]^ |  |  |
|  | Northeast Siberia | 68°N-70°N,  155°E-162°E | Permafrost | *Methylobacter* | ^[91]^ |  |  |
|  | Irtysh Basin, West Siberia | 56°51′N, 82°50′E | Floodplain | *Methylovulum* | ^[92]^ |  |  |
|  | Mukhrinskaya, Irtysh Basin, West Siberia | 65°35′N, 72°41′E | Floodplain | *Methylovulum* | ^[93]^ |  |  |
|  | River Mukhrinskaya, West Siberia | 60°53′N, 68°42′E | Floodplain | *Methylobacter* | ^[94]^ |  |  |
|  | Bykovsky Peninsula, Northeastern Siberia | 71°46′N, 129°11′E | Thermokarst Lagoon | *Methylobacter*, *Crenothrix* | ^[95]^ |  |  |
|  | Around Igarka, Russia | 67.465°N, 86.578°E | Lake | *Methylobacter*, *Crenothrix* | ^[96]^ |  |  |
|  | Boreal Zone | 56°51′N, 82°50′E | Peatland | *Methylocystis*,  *Methylocella*, *Methylocapsa* | ^[97]^ |  |  |
|  | Bakchar Bog, West Siberia | 56°N, 82°E | Peat Bog | *Methylocella* | ^[98]^ |  |  |
|  | Bakchar Bog, West Siberia | 56°51′N, 82°50′E | Peat Bog | *Methylocystis* | ^[62]^**^a^** |  |  |
|  | Tomsk Region, West Siberia | 56°51′N, 82°50′E | Peat Bog | *Methylocystis* | ^[99]^ |  |  |
|  | Island Valaam, Karelia, North-Eastern Russia | 56°10′N, 85°00′E | Peat Bog | *Methylomonas* | ^[100]^ |  |  |
|  | Torfjanoye, European North Russia; West Siberia | 65°01′N, 35°44′E;  56°51′N, 82°51′E | Peat Bog | *Methyloferula* | ^[101]^ |  |  |
|  | West Siberia and the European plain of Russia. | 56°01′N, 32°12′E;  56°N, 85°E;  52°53′N, 82°50′E; | Peat Bog | *Methylocystis*, *Methylosinus* | ^[102]^ |  |  |
|  | Bakchar, West Siberia | 56°N, 82°E | Acidic Peat Bog | *Methylocapsa* | ^[103]^ |  |  |
|  | Acidic Sphagnum Peat Bogs, Siberia | 56°01′N, 32°12′E;  56°N, 85°E;  52°53′N, 82°50′E; | Peat Bog | *Methylocella* | ^[104]^ |  |  |
|  | Acidic Sphagnum Peat Bogs, Siberia | 55.5°N-58.5°N, 31°E-38°E | Peat Bog | *Methylocystis, Methylocella, Methylocapsa* | ^[105]^ |  |  |
|  | Acidic Sphagnum Tundra Peatland in Northern Russia | 67°20′N, 62°44′E | Tundra Peatland | *Methylocella* | ^[106]^ |  |  |
|  | Tundra Wetland, Northern Russia | 67°20′N, 62°44′E | Tundra Peatland | *Methylocella* | ^[107]^ |  |  |
|  | Tundra Region, Russia | 67°30′N, 64°00′E | Tundra | *Methylobacter* | ^[108]^ |  |  |
|  | Tundra Region, Russia | 67°30′N, 64°00′E | Tundra | *Methylobacter* | ^[109]^ |  |  |
|  | Russian Arctic Tundra | 68°00′N-161°00′E | Tundra | *Methylobacter* | ^[110]^ |  |  |
|  | Russian NorthernTaiga and Subarctic Tundra | 66°39′N, 66°24′E | Tundra | *Methylobacter, Methylomonas* | ^[111]^ |  |  |
|  | Tal’nik station | 67°20′N, 62°44′E | Tundra | *Methylobacter, Methylocella* | ^[112]^ |  |  |
| **Spain** | Xistral | 42°48′N, 8°6′W | Peatland | *Methylocystis* | ^[46]^**^b^** | 1 | *Methylocystis* (1)=100% |
| **Sweden** | Stor-Åmyran,  Rjörnmyran | 63°44′N, 20°06′E  64°20′N, 18°18′E | Peatland | *Methylomonas*, *Methylosinus* | ^[113]^ | 3 | *Methylomonas*+*Methylosinus*(1/3)=33.33%;  *Methylocella*(1/3)=33.33% |
|  | Hornavan,  Uddjaure | 66°13′N, 17°78′E;  65°60′N, 17°48′E | Peatland | *Methylocella* | ^[48]^**^c^** |  |  |
|  | Stordalen Mire | 68°21′N, 19°03′E | Palsa,Bog,Fen | *Methylocystis* | ^[114]^ |  |  |
| **United Kingdom** | Moorhouse Reserve | 54°41'N, 2°23'W | Peatland | *Methylosinus* | ^[115]^ | 6 | *Methylocystis* (4/6)=66.70% |
|  | Moorhouse Reserve | 54°41'N, 2°23'W | Peatland | *Methylocystis* | ^[116]^ |  |  |
|  | Moorhouse,Auchencorth, Plynlimon, Caithness, Clara Bog | 54°42'N, 2°18'W  55°47′N, 3°14′W  52°26′N, 3°46′W  58°08′N, 0°36′W  53°19′N, 7°39′W | Peatland | *Methylocystis* | ^[46]^**^b^** |  |  |
|  | North Pennines of England | 54°65′N, 2°45′W | Peatland | *Methylocystis* | ^[117]^ |  |  |
|  | Suffolk | 52°59′N, 3°50′W | Peatland | *Methylocystis* | ^[118]^ |  |  |
|  | Moor House,  Wales | 54°42′N, 2°18′W, 52°26′N, 3°46′W | Peatland | *Methylocella* | ^[48]^**^c^** |  |  |
| **United States** | Lake Qalluuraq, Brooks Range in Alaska | 70°23′N, 157°21′W | Tundra Lake | *Methylobacter, Methylomonas, Methylosoma* | ^[119]^ | 9 | *Methylobacter* (5/9)=55.56% |
|  | Lake Qalluuraq, Brooks Range in Alaska | 70°23′N, 157°21′W | Tundra Lake | *Methylobacter* | ^[120]^ |  |  |
|  | Alaska | 64°51′N, 147°43′W | Permafrost | *Methylocapsa* | ^[10]^**^c^** |  |  |
|  | Alaska | 63°N-68°N,  151°E-146°E | Peatland, Lake and Pond | *Methylobacter* | ^[86]^**^a^** |  |  |
|  | Marcell Experimental Forest | 47°30′N, 93°27′W | Peatland | *Methylocystis*, *Methylomonas* | ^[121]^ |  |  |
|  | Chicapp Bog,  Michigan Hollow | 47°32′N, 93°28′W | Peatland | *Methylocystis*, *Methylosinus* | ^[122]^ |  |  |
|  | Lake Washington in Seattle | 47°37′N, 122°14′W | Lake | *Methylobacter* | ^[123]^ |  |  |
|  | Cottonwood Lake,  Old Woman Creek | 47°05′N: 99°06′W;  41°22′N 82°30′W | Lake | *Methylobacter*, *Methylomonas* | ^[124]^ |  |  |
|  | Ithaca, New York | 42°28′N, 76°26′W | Swamp | *Methylomonas* | ^[125]^ |  |  |
| **Total number of MOB studies** | | | | | | **135** |  |

***^.^** Countries were sorted alphabetically.

**^a.^** Counted twice, as the study collected samples from two countries; **^b.^** Counted three times, as the study collected samples from three countries. **^c.^** Counted four times, as the study collected samples from four countries.Consequently, the total number of studies related to aerobic methanotrophs is 135. For studies reporting multiple co-dominant MOB genera, the first-listed genus was used for country-level frequency calculations; each study was counted only once to avoid double-counting.

**Table S2** Reference list of studies on anaerobic methanotrophs by country, including the predominant anaerobic methanotroph genera reported in each study, together with the most frequently reported predominant genus in the reviewed studies from each country (frequency of studies).

| **Country*** | **Study site** | **Location** | **Wetland type** | **Dominant anaerobic methanotrophs in each study** | **Ref.** | **Number of studies in each country** | **Percentage of studies that dominant AOM microbes were detected in each country** |
| --- | --- | --- | --- | --- | --- | --- | --- |
| **China** | Zoige Reserve, 3433 m | 33°57′N, 102°50′E | Alpine | ANME-2d | ^[126]^ | 11 | NC10 (7/11)=63.6%;  ANME-2d (4/11)=36.4% |
|  | Zoige Reserve, 3433 m | 33°57′N, 102°50′E | Alpine | ANME-2d, NC10 | ^[127]^ |  |  |
|  | Zoige Reserve, 3433 m | 33°57'N, 102°50′E | Alpine | Z-aquifer Subcluster, NC10 | ^[128]^ |  |  |
|  | Zoige Reserve, 3433 m | 33°56′N, 102°52′E | Alpine | NC10 | ^[129]^ |  |  |
|  | Gahai Lake 1, Lake 2 3460 m, 4080 m | 36°58′N, 100°36′E;  37°08′N, 97°35′E | Plateau Lake | NC10 | ^[130]^ |  |  |
|  | Yunnan Plateau, 1273-2555 m | 23.43°N-28.68°N, 99.28°N-103.99°E | Freshwater | NC10 | ^[20]^**^a^** |  |  |
|  | Tianchi ; Songhua River | 43°54′N, 88°08′E;  44°04′N-46°40′N, 125°42′E-130°10′E | Alpine | NC10 | ^[131]^ |  |  |
|  | Daxing'an Mountains | 52°28′N-53°26′N, 122°12′E-122°21′E | Permafrost | NC10 | ^[132]^ |  |  |
|  | Lake Fuxian, 1750 m | 24.6°00′N, 102.9°00′E | Plateau Lake | ANME-2d, NC10 | ^[133]^ |  |  |
|  | Erhai Lake, 1972 m;  Dianchi Lake,1886 m | 25°36′N-25°58′N，100°05′E-100°18′E;  24°40′N-25°02′N，102°36′E-102°47′E | Lake | NC10 | ^[134]^ |  |  |
|  | Maqu, 3450 m | 33°40′N, 101°53′E | Alpine Swamp | ANME-2d | ^[37]^**^a^** |  |  |
| **Denmark** | Jutland | 56°08′N, 9°32′E | Freshwater | ANME-2d | ^[135]^ | 1 | ANME-2d (1)=100% |
| **Finland** | Pojo Bay Estuarine System | 60.0°N, 23.5°E | Estuarine | ANME-2a/b | ^[136]^ | 2 | ANME-2a/b (1/2)=50.0%；  ANME-2d+NC10 (1/2)=50.0% |
|  | Lake Kevätön and Lake Pöljä | 62°31′N, 29°22′E  62°02′N, 29°59′E | Lake | ANME-2d, NC10 | ^[50]^ |  |  |
| **Germany** | Heiligensee-Hütelmoor Glacial Channel Wetland Reserve | 52°37′N, 13°14′E | Marsh | ANME-2d | ^[60]^**^a^** | 4 | ANME-2d (2/4)=50.0%; NC10 (2/4)=50.0% |
|  | Lake Constance | 47°35′N, 9°25′E | Lake | NC10 | ^[137]^ |  |  |
|  | Lake Constance | 47°35′N, 9°25′E | Lake | NC10 | ^[138]^ |  |  |
|  | Heiligensee-Hütelmoor Glacial Channel Wetland Reserve | 52°36′N, 13°14′E | Marsh | ANME-2d | ^[61]^**^a^** |  |  |
| **Netherlands** | Brunssummerheide | 50°56′N, 5°59′E | Marsh | NC10 | ^[139]^ | 6 | ANME-2d (2/6)=33.3%;  NC10 (4/6)=66.6% |
|  | Twentekanaal and Ooijpolder | 52°11′N, 6°25′E; 51°51′N, 5°55′E | Freshwater | NC10 | ^[140]^ |  |  |
|  | Ooijpolder | 51°51′N, 5°55′E | Floodplain | NC10 | ^[141]^ |  |  |
|  | Twentekanaal | 52°11′N, 6°24′E | Freshwater | ANME-2d | ^[142]^ |  |  |
|  | Twentekanaal | 52°11′N, 6°24′E | Freshwater | ANME-2d | ^[143]^ |  |  |
|  | Twentekanaal | 52°11′N, 6°24′E | Freshwater | NC10 | ^[144]^ |  |  |
| **Russia** | Laptev Sea Marginal Ice Zone | 72°00′N-81°00′N, 100°00′E-145°00′E | Permafrost | ANME-2d | ^[145]^ | 3 | ANME-2d (2/3)=66.7%; NC10(1/3)=33.3% |
|  | Posolsk Bank | 52.03°N, 105.84°E | Littoral | ANME-2d | ^[146]^ |  |  |
|  | Lena Delta Cryolithozone Megadelta | 72°24′N, 126°41′E | Permafrost | NC10 | ^[95]^**^a^** |  |  |
| **Switzerland** | Lake Cadagno Meromictic | 46°33′N, 8°43′E | Alpine Lake | ANME-2d | ^[147]^ | 3 | ANME-2d (3/3)=100% |
|  | Lake Cadagno Meromictic | 46°33′N, 8°43′E | Alpine Lake | ANME-2d | ^[148]^ |  |  |
|  | Lake Cadagno | 46°33′N, 8°42′E | Alpine Lake | ANME-2d | ^[149]^ |  |  |
| **United States** | Michigan Hollow Fen | 45°25′N, 84°05′W | Peatland | NC10 | ^[150]^ | 3 | ANME2 (2/3)=66.7%;  NC10 (1/3)=33.3% |
|  | Vault Lake Thermokarst | 64°51′N, 147°43′W | Permafrost lake | ANME-2d | ^[151]^ |  |  |
|  | Barrow Environmental Observatory | 71.30°N, 156.59°W | Permafrost Lake | ANME2 | ^[152]^ |  |  |
| **Total number of AOM studies in cold wetlands** | | | | | | **33** |  |

*. Countries were sorted alphabetically. **^a.^** Counted twice because both aerobic and anaerobic methanotrophic genera were reported in the study; the study was previously counted in the statistics for aerobic methanotroph–related research and is counted again here as an anaerobic methanotroph. Accordingly, the total number of studies related to anaerobic methanotrophs is recorded as 33.

**References**

1.	Roldán DM, Menes RJ Characterisation of ‘Candidatus Methylobacter titanis’ sp. nov., a putative novel species of Methylobacter clade 2 and their distribution in sediments of freshwater lakes in maritime Antarctica*.* *Antonie Van Leeuwenhoek*, 2023. **116**: 721-38. <https://doi.org/10.1007/s10482-023-01840-1>

2.	Roldán DM, Carrizo D, Sánchez-García L *et al.* Diversity and effect of increasing temperature on the activity of methanotrophs in sediments of fildes Peninsula freshwater lakes, King George Island, Antarctica*.* *Frontiers in Microbiology*, 2022. **13**: 822552. <https://doi.org/10.3389/fmicb.2022.822552>

3.	Bowman JP, McCammon SA, Skerrat JH Methylosphaera hansonii gen. nov., sp. nov., a psychrophilic, group I methanotroph from Antarctic marine-salinity, meromictic lakes*.* *Microbiology*, 1997. **143**: 1451-9. <https://doi.org/10.1099/00221287-143-4-1451>

4.	Kip N, Van Winden JF, Pan Y *et al.* Global prevalence of methane oxidation by symbiotic bacteria in peat-moss ecosystems*.* *Nature Geoscience*, 2010. **3**: 617-21. <https://doi.org/10.1038/NGEO939>

5.	Kip N, Fritz C, Langelaan E *et al.* Methanotrophic activity and diversity in different Sphagnum magellanicum dominated habitats in the southernmost peat bogs of Patagonia*.* *Biogeosciences*, 2012. **9**: 47-55. <https://doi.org/10.5194/bg-9-47-2012>

6.	Altshuler I, Raymond-Bouchard I, Magnuson E *et al.* Unique high Arctic methane metabolizing community revealed through in situ 13CH4-DNA-SIP enrichment in concert with genome binning*.* *Scientific Reports*, 2022. **12**: 1160. <https://doi.org/10.1038/s41598-021-04486-z>

7.	Barbier BA, Dziduch I, Liebner S *et al.* Methane-cycling communities in a permafrost-affected soil on Herschel Island, Western Canadian Arctic: active layer profiling of mcrA and pmoA genes*.* *FEMS microbiology ecology*, 2012. **82**: 287-302. <https://doi.org/10.1111/j.1574-6941.2012.01332.x>

8.	Pacheco-Oliver M, McDonald IR, Groleau D *et al.* Detection of methanotrophs with highly divergent pmoA genes from Arctic soils*.* *FEMS Microbiology Letters*, 2002. **209**: 313-9. <https://doi.org/10.1016/s0378-1097(02)00568-2>

9.	Szylit A, Christaki U, Barret M *et al.* Spatial Heterogeneity in Methane Biogeochemistry and Prokaryotic Community Structure in Sub‐Arctic Waterbodies in Northern Canada*.* *Environmental Microbiology*, 2025. **27**: e70210. <https://doi.org/10.1111/1462-2920.70210>

10.	Wang H, Lindemann E, Liebmann P *et al.* Methane-cycling microbiomes in soils of the pan-Arctic and their response to permafrost degradation*.* *Communications Earth & Environment*, 2025. **6**: 748. <https://doi.org/10.1038/s43247-025-02765-5>

11.	Crevecoeur S, Vincent WF, Comte J *et al.* Diversity and potential activity of methanotrophs in high methane-emitting permafrost thaw ponds*.* *PLoS One*, 2017. **12**: e0188223. <https://doi.org/10.1371/journal.pone.0188223>

12.	Wang XC, Kelong, Wang H, Zhang N *et al.* Community characteristics of methane-oxidizing bacteria in three types of alpine wetlands around Qinghai Lake*.* *Microbiology China*, 2023. **50**: 4357-71. <https://doi.org/10.13344/j.microbiol.china.230079>

13.	Yun J, Ju Y, Deng Y *et al.* Bacterial community structure in two permafrost wetlands on the Tibetan Plateau and Sanjiang Plain, China*.* *Microbial ecology*, 2014. **68**: 360-9. <https://doi.org/10.1007/s00248-014-0415-4>

14.	Zhang L, Dumont MG, Bodelier PL *et al.* DNA stable-isotope probing highlights the effects of temperature on functionally active methanotrophs in natural wetlands*.* *Soil Biology and Biochemistry*, 2020. **149**: 107954. <https://doi.org/10.1016/j.soilbio.2020.107954>

15.	Yun J, Yu Z, Li K *et al.* Diversity, abundance and vertical distribution of methane-oxidizing bacteria (methanotrophs) in the sediments of the Xianghai wetland, Songnen Plain, northeast China*.* *Journal of soils and sediments*, 2013. **13**: 242-52. <https://doi.org/10.1007/s11368-012-0610-1>

16.	Liu X, Shi Y, Yang T *et al.* Distinct co-occurrence relationships and assembly processes of active methane-oxidizing bacterial communities between paddy and natural wetlands of northeast China*.* *Frontiers in microbiology*, 2022. **13**: 809074. <https://doi.org/10.3389/fmicb.2022.809074>

17.	Wang Q Y WN, Liu Y, et al Microbial community structure of soil methanogens and methanotrophs during degradation and restoration of reed Wetlands in the Songnen Plain[J]*.* *Huan jing ke xue= Huanjing kexue*, 2021. **42**: 4968-76. <https://doi.org/10.13227/j.hjkx.202102172>

18.	Yun J, Zhang H, Deng Y *et al.* Aerobic methanotroph diversity in Sanjiang wetland, Northeast China*.* *Microbial ecology*, 2015. **69**: 567-76. <https://doi.org/10.1007/s00248-014-0506-2>

19.	Kang Y, Wu H, Guan Q *et al.* Earthworms and warming alter methane uptake and methane-cycling microbial community in meadow soil*.* *Soil Ecology Letters*, 2024. **6**: 240255. <https://doi.org/10.1007/s42832-024-0255-1>

20.	Liu Y, Zhang J, Zhao L *et al.* Aerobic and nitrite-dependent methane-oxidizing microorganisms in sediments of freshwater lakes on the Yunnan Plateau*.* *Applied Microbiology and Biotechnology*, 2015. **99**: 2371-81. <https://doi.org/10.1007/s00253-014-6141-5>

21.	Yun J, Crombie AT, Ul Haque MF *et al.* Revealing the community and metabolic potential of active methanotrophs by targeted metagenomics in the Zoige wetland of the Tibetan Plateau*.* *Environmental Microbiology*, 2021. **23**: 6520-35. <https://doi.org/10.1111/1462-2920.15697>

22.	Yun J, Ma A, Li Y *et al.* Diversity of methanotrophs in Zoige wetland soils under both anaerobic and aerobic conditions*.* *Journal of Environmental Sciences*, 2010. **22**: 1232-8. <https://doi.org/10.1016/S1001-0742(09)60243-6>

23.	Deng Y, Liang C, Zhu X *et al.* Methylomonadaceae was the active and dominant methanotroph in Tibet lake sediments*.* *ISME communications*, 2024. **4**: ycae032. <https://doi.org/10.1093/ismeco/ycae032>

24.	Deng Y, Liu Y, Dumont M *et al.* Salinity affects the composition of the aerobic methanotroph community in alkaline lake sediments from the Tibetan Plateau*.* *Microbial ecology*, 2017. **73**: 101-10. <https://doi.org/10.1007/s00248-016-0879-5>

25.	Fang J, Adams JM, Deng Y *et al.* Propagule limitation affects the response of soil methane oxidizer community to increased salinity*.* *Geoderma*, 2022. **426**: 116082. <https://doi.org/10.1016/j.geoderma.2022.116082>

26.	Ji W, Ren L, Zhou Z *et al.* Characteristics of methanotrophic communities and their physicochemical driving mechanisms in estuarine and nearshore wetlands of Qinghai Lake*.* *iScience*, 2025. **28**. <https://doi.org/10.1016/j.isci.2025.113040>

27.	Deng Y, Cui X, Lüke C *et al.* Aerobic methanotroph diversity in R iganqiao peatlands on the Q inghai–T ibetan P lateau*.* *Environmental microbiology reports*, 2013. **5**: 566-74. <https://doi.org/10.1111/1758-2229.12046>

28.	Deng Y, Cui X, Hernandez M *et al.* Microbial diversity in hummock and hollow soils of three wetlands on the Qinghai-Tibetan Plateau revealed by 16S rRNA pyrosequencing*.* *PloS one*, 2014. **9**: e103115. <https://doi.org/10.1371/journal.pone.0103115>

29.	Deng Y, Cui X, Dumont MG Identification of active aerobic methanotrophs in plateau wetlands using DNA stable isotope probing*.* *FEMS Microbiology Letters*, 2016. **363**(16): fnw168. <https://doi.org/10.1093/femsle/fnw168>

30.	Liu A, Yin W, Ma D *et al.* Vertical distribution patterns and potential activities of methanogenic and methanotrophic communities in permafrost peatlands of Greater Khingan Mountains*.* *Ecological Indicators*, 2025. **175**: 113539. <https://doi.org/10.1016/j.ecolind.2025.113539>

31.	Tian W, Wang H, Xiang X *et al.* Water table level controls methanogenic and methanotrophic communities and methane emissions in a Sphagnum-dominated peatland*.* *Microbiol Spectr*, 2023. **11**: e0199223. <https://doi.org/10.1128/spectrum.01992-23>

32.	Zhang Y, Cui M, Duan J *et al.* Abundance, rather than composition, of methane‐cycling microbes mainly affects methane emissions from different vegetation soils in the Zoige alpine wetland*.* *MicrobiologyOpen*, 2019. **8**: e00699. <https://doi.org/10.1002/mbo3.699>

33.	Yun J, Zhuang G, Ma A *et al.* Community structure, abundance, and activity of methanotrophs in the Zoige wetland of the Tibetan Plateau*.* *Microbial ecology*, 2012. **63**: 835-43. <https://doi.org/10.1007/s00248-011-9981-x>

34.	Zhang L, Adams JM, Dumont MG *et al.* Distinct methanotrophic communities exist in habitats with different soil water contents*.* *Soil Biology and Biochemistry*, 2019. **132**: 143-152. <https://doi.org/https://doi.org/10.1016/j.soilbio.2019.02.007>

35.	Cao P, Wang Y, Wang Z *et al.* Diversity and community assembly mechanisms of soil methanotrophs in typical ecotypes of the Mitika alpine wetland in northern Xizang*.* *Frontiers in Microbiology*, 2025. **16**: 1731213. <https://doi.org/10.3389/fmicb.2025.1731213>

36.	Tong Y, Liu J, Zhu X *et al.* Methane oxidation in alpine meadow and wetland soils on the Qinghai-Tibet Plateau: Roles of USCγ and conventional methanotrophs*.* *Applied Soil Ecology*, 2025. **211**: 106104. <https://doi.org/10.1016/j.apsoil.2025.106104>

37.	Mo Y, Qi X-e, Li A *et al.* Active methanotrophs in suboxic alpine swamp soils of the Qinghai–Tibetan Plateau*.* *Frontiers in Microbiology*, 2020. **11**: 580866. <https://doi.org/10.3389/fmicb.2020.580866>

38.	Xing T, Liu P, Ji M *et al.* Sink or source: alternative roles of glacier foreland meadow soils in methane emission is regulated by glacier melting on the Tibetan plateau*.* *Frontiers in Microbiology*, 2022. **13**: 862242. <https://doi.org/10.3389/fmicb.2022.862242>

39.	Zhu X, Deng Y, Hernández M *et al.* Distinct responses of soil methanotrophy in hummocks and hollows to simulated glacier meltwater and temperature rise in Tibetan glacier foreland*.* *Science of The Total Environment*, 2023. **862**: 160888. <https://doi.org/10.1016/j.scitotenv.2022.160888>

40.	Zhu X, Deng Y, Liu Y Methylocystis dominates methane oxidation in glacier foreland soil at elevated temperature*.* *FEMS Microbiology Letters*, 2024. **371**: fnae011. <https://doi.org/10.1093/femsle/fnae011>

41.	Huang M, Mu G, Mai F *et al.* Methane cycling in typical emerging proglacial lakes on the Tibetan Plateau: Insights into the metabolic mechanisms mediated by microorganisms*.* *Water Research*, 2025. **280**: 123533. <https://doi.org/10.1016/j.watres.2025.123533>

42.	Christiansen JR, Romero AJB, Jørgensen NO *et al.* Methane fluxes and the functional groups of methanotrophs and methanogens in a young Arctic landscape on Disko Island, West Greenland*.* *Biogeochemistry*, 2015. **122**: 15-33. <https://doi.org/10.1007/s10533-014-0026-7>

43.	Knudsen K, Sereika M, Jensen T *et al.* A national baseline for methane sink habitats and methanotroph diversity*.* *bioRxiv*, 2026. **649**: 971–9. <https://doi.org/10.1038/s41586-025-09794-2>

44.	Siljanen HM, Saari A, Krause S *et al.* Hydrology is reflected in the functioning and community composition of methanotrophs in the littoral wetland of a boreal lake*.* *FEMS microbiology ecology*, 2011. **75**: 430-45. <https://doi.org/10.1111/j.1574-6941.2010.01015.x>

45.	Siljanen HM, Saari A, Bodrossy L *et al.* Seasonal variation in the function and diversity of methanotrophs in the littoral wetland of a boreal eutrophic lake*.* *FEMS microbiology ecology*, 2012. **80**: 548-55. <https://doi.org/10.1111/j.1574-6941.2012.01321.x>

46.	Chen Y, Dumont MG, Neufeld JD *et al.* Revealing the uncultivated majority: combining DNA stable‐isotope probing, multiple displacement amplification and metagenomic analyses of uncultivated Methylocystis in acidic peatlands*.* *Environmental Microbiology*, 2008. **10**: 2609-22. <https://doi.org/10.1111/j.1462-2920.2008.01683.x>.

47.	Jaatinen K, Tuittila E-S, Laine J *et al.* Methane-oxidizing bacteria in a Finnish raised mire complex: effects of site fertility and drainage*.* *Microbial ecology*, 2005. **50**: 429-39. <https://doi.org/10.1007/s00248-005-9219-x>

48.	Rahman MT, Crombie A, Chen Y *et al.* Environmental distribution and abundance of the facultative methanotroph Methylocella*.* *The ISME Journal*, 2010. **5**: 1061-6. <https://doi.org/10.1038/ismej.2010.190>

49.	Putkinen A, Larmola T, Tuomivirta T *et al.* Peatland succession induces a shift in the community composition of Sphagnum-associated active methanotrophs*.* *FEMS Microbiology Ecology*, 2014. **88**: 596-611. <https://doi.org/10.1111/1574-6941.12327>

50.	Rissanen AJ, Karvinen A, Nykänen H *et al.* Effects of alternative electron acceptors on the activity and community structure of methane-producing and consuming microbes in the sediments of two shallow boreal lakes*.* *FEMS Microbiology Ecology*, 2017. **93**. <https://doi.org/10.1093/femsec/fix078>

51.	Khanongnuch R, Mangayil R, Rissanen Antti J Conversion of methane to organic acids is a widely found trait among gammaproteobacterial methanotrophs of freshwater lake and pond ecosystems*.* *Microbiology Spectrum*, 2023. **11**: e01742-23. <https://doi.org/10.1128/spectrum.01742-23>

52.	Rissanen AJ, Buck M, Peura S 16S rRNA gene sequences of Candidatus Methylumidiphilus (Methylococcales), a putative methanotrophic genus in lakes and ponds*.* *AQUATIC MICROBIAL ECOLOGY*, 2022. **88**: 25-30. <https://doi.org/10.3354/ame01983>

53.	Rissanen AJ, Mangayil R, Svenning MM *et al.* Draft genome sequence data of methanotrophic Methylovulum psychrotolerans strain S1L and Methylomonas paludis strain S2AM isolated from hypoxic water column layers of boreal lakes*.* *Data in Brief*, 2021. **38**: 107364. <https://doi.org/10.1016/j.dib.2021.107364>

54.	Rissanen AJ, Saarenheimo J, Tiirola M *et al.* Gammaproteobacterial methanotrophs dominate methanotrophy in aerobic and anaerobic layers of boreal lake waters*.* *Aquatic Microbial Ecology*, 2018. **81**: 257-76. <https://doi.org/10.3354/ame01874>

55.	Khanongnuch R, Mangayil R, Svenning MM *et al.* Characterization and genome analysis of a psychrophilic methanotroph representing a ubiquitous Methylobacter spp. cluster in boreal lake ecosystems*.* *ISME communications*, 2022. **2**: 85. <https://doi.org/10.1038/s43705-022-00172-x>

56.	Rissanen AJ, Mangayil R, Khanongnuch R Genome of Methylomonas sp. AM2-LC, representing a methanotrophic bacterial species isolated from water column of a boreal, oxygen-stratified lake*.* *Frontiers in Genetics*, 2024. **15**: 1440435. <https://doi.org/10.3389/fgene.2024.1440435>

57.	Oshkin IY, Miroshnikov KK, Dedysh SN Draft Genome Sequence of Methylocystis heyeri H2T, a Methanotroph with Habitat-Specific Adaptations, Isolated from a Peatland Ecosystem*.* *Microbiology resource announcements*, 2019. **8**: 10-1128. <https://doi.org/10.1128/mra.00454-19>

58.	Dedysh SN, Belova SE, Bodelier PL *et al.* Methylocystis heyeri sp. nov., a novel type II methanotrophic bacterium possessing ‘signature’fatty acids of type I methanotrophs*.* *International journal of systematic and evolutionary microbiology*, 2007. **57**: 472-9. <https://doi.org/10.1099/ijs.0.64623-0>

59.	Belova SE, Baani M, Suzina NE *et al.* Acetate utilization as a survival strategy of peat‐inhabiting Methylocystis spp*.* *Environmental Microbiology Reports*, 2011. **3**: 36-46. <https://doi.org/10.1111/j.1758-2229.2010.00180.x>

60.	Gutekunst CN, Liebner S, Jenner A-K *et al.* Effects of brackish water inflow on methane-cycling microbial communities in a freshwater rewetted coastal fen*.* *Biogeosciences*, 2022. **19**: 3625-48. <https://doi.org/10.5194/bg-19-3625-2022>

61.	Wen X, Unger V, Jurasinski G *et al.* Predominance of methanogens over methanotrophs in rewetted fens characterized by high methane emissions*.* *Biogeosciences*, 2018. **15**: 6519-36. <https://doi.org/10.5194/bg-15-6519-2018>

62.	Dedysh SN, Dunfield PF, Derakshani M *et al.* Differential detection of type II methanotrophic bacteria in acidic peatlands using newly developed 16S rRNA-targeted fluorescent oligonucleotide probes*.* *FEMS Microbiology Ecology*, 2003. **43**: 299-308. <https://doi.org/10.1111/j.1574-6941>

63.	Dunfield PF, Khmelenina VN, Suzina NE *et al.* Methylocella silvestris sp. nov., a novel methanotroph isolated from an acidic forest cambisol*.* *International Journal of Systematic and Evolutionary Microbiology*, 2003. **53**: 1231-9. <https://doi.org/10.1099/ijs.0.02481-0>

64.	Chen Y, Crombie A, Rahman MT *et al.* Complete genome sequence of the aerobic facultative methanotroph Methylocella silvestris BL2*.* *Journal of bacteriology*, 2010. **192**: 3840-3841. <https://doi.org/10.1128/jb.00506-10>

65.	Bussmann I, Horn F, Hoppert M *et al.* Methylomonas albis sp. nov. and Methylomonas fluvii sp. nov.: two cold-adapted methanotrophs from the river Elbe and emended description of the species Methylovulum psychrotolerans*.* *Systematic and Applied Microbiology*, 2021. **44**: 126248. <https://doi.org/10.1016/j.syapm.2021.126248>

66.	Dunfield PF, Belova SE, Vorob'ev AV *et al.* Methylocapsa aurea sp. nov., a facultative methanotroph possessing a particulate methane monooxygenase, and emended description of the genus Methylocapsa*.* *International journal of systematic and evolutionary microbiology*, 2010. **60**: 2659-64. <https://doi.org/10.1099/ijs.0.020149-0>

67.	Narihiro T, Hori T, Nagata O *et al.* The impact of aridification and vegetation type on changes in the community structure of methane-cycling microorganisms in Japanese wetland soils*.* *Bioscience, biotechnology, and biochemistry*, 2011. **75**: 1727-34. <https://doi.org/10.1271/bbb.110265>

68.	Bodelier PL, Meima-Franke M, Zwart G *et al.* New DGGE strategies for the analyses of methanotrophic microbial communities using different combinations of existing 16S rRNA-based primers*.* *FEMS microbiology ecology*, 2005. **52**: 163-174. <https://doi.org/10.1016/j.femsec.2004.11.004>

69.	Kip N, Dutilh BE, Pan Y *et al.* Ultra‐deep pyrosequencing of pmoA amplicons confirms the prevalence of Methylomonas and Methylocystis in Sphagnum mosses from a Dutch peat bog*.* *Environmental microbiology reports*, 2011. **3**: 667-73. <https://doi.org/10.1111/j.1758-2229.2011.00260.x>

70.	Kip N, Ouyang W, van Winden J *et al.* Detection, isolation, and characterization of acidophilic methanotrophs from Sphagnum mosses*.* *Applied and environmental microbiology*, 2011. **77**: 5643-54. <https://doi.org/10.1128/AEM.05017-11>

71.	Raghoebarsing AA, Smolders AJ, Schmid MC *et al.* Methanotrophic symbionts provide carbon for photosynthesis in peat bogs*.* *Nature*, 2005. **436**: 1153-1156. <https://doi.org/10.1038/nature03802>

72.	Svenning MM, Hestnes AG, Wartiainen I *et al.* Genome Sequence of the Arctic Methanotroph Methylobacter tundripaludum SV96*.* *JOURNAL OF BACTERIOLOGY*, 2011. **193**: 6418-9. <https://doi.org/10.1128/JB.05380-11>

73.	Wartiainen I, Hestnes AG, McDonald IR *et al.* Methylocystis rosea sp. nov., a novel methanotrophic bacterium from Arctic wetland soil, Svalbard, Norway (78 N)*.* *International journal of systematic and evolutionary microbiology*, 2006. **56**: 541-7. <https://doi.org/10.1099/ijs.0.63912-0>

74.	Wartiainen I, Hestnes AG, McDonald IR *et al.* Methylobacter tundripaludum sp. nov., a methane-oxidizing bacterium from Arctic wetland soil on the Svalbard islands, Norway (78 N)*.* *International Journal of Systematic and Evolutionary Microbiology*, 2006. **56**: 109-13. <https://doi.org/10.1099/ijs.0.63728-0>

75.	Wartiainen I, Hestnes AG, Svenning MM Methanotrophic diversity in high arctic wetlands on the islands of Svalbard (Norway)-denaturing gradient gel electrophoresis analysis of soil DNA and enrichment cultures*.* *Canadian journal of microbiology*, 2003. **49**: 602-12. <https://doi.org/10.1139/w03-080>

76.	Patil SK, Islam T, Tveit A *et al.* Targeting methanotrophs and isolation of a novel psychrophilic Methylobacter species from a terrestrial Arctic alkaline methane seep in Lagoon Pingo, Central Spitsbergen (78° N)*.* *Antonie van Leeuwenhoek*, 2024. **117**: 60. <https://doi.org/10.1007/s10482-024-01953-1>

77.	Tveit A, Schwacke R, Svenning MM *et al.* Organic carbon transformations in high-Arctic peat soils: key functions and microorganisms*.* *The ISME Journal*, 2012. **7**: 299-311. <https://doi.org/10.1038/ismej.2012.99>

78.	Dedysh SN, Didriksen A, Danilova OV *et al.* Methylocapsa palsarum sp. nov., a methanotroph isolated from a subArctic discontinuous permafrost ecosystem*.* *International journal of systematic and evolutionary microbiology*, 2015. **65**: 3618-24. <https://doi.org/10.1099/ijsem.0.000465>

79.	Liebner S, Svenning MM Environmental transcription of mmoX by methane-oxidizing Proteobacteria in a subarctic palsa peatland*.* *Applied and Environmental Microbiology*, 2013. **79**: 701-6. <https://doi.org/10.1128/aem.02292-12>

80.	Graef C, Hestnes AG, Svenning MM *et al.* The active methanotrophic community in a wetland from the High Arctic*.* *Environmental microbiology reports*, 2011. **3**: 466-72. <https://doi.org/10.1111/j.1758-2229.2010.00237.x>

81.	Miroshnikov KK, Didriksen A, Naumoff DG *et al.* Draft genome sequence of Methylocapsa palsarum NE2T, an obligate methanotroph from subarctic soil*.* *Genome announcements*, 2017. **5**: 504-17. <https://doi.org/10.1128/genomea.00504-17>

82.	Tveit Alexander T, Urich T, Svenning Mette M Metatranscriptomic Analysis of Arctic Peat Soil Microbiota*.* *Applied and Environmental Microbiology*, 2014. **80**: 5761-72. <https://doi.org/10.1128/AEM.01030-14>

83.	Rainer EM, Seppey CV, Hammer C *et al.* The influence of above-ground herbivory on the response of arctic soil methanotrophs to increasing CH4 concentrations and temperatures*.* *Microorganisms*, 2021. **9**: 2080. <https://doi.org/10.3390/microorganisms9102080>

84.	Jensen S, Siljanen HMP, Dörsch P Activity and abundance of methanotrophic bacteria in a northern mountainous gradient of wetlands*.* *Environmental Microbiology Reports*, 2023. **15**: 206-15. <https://doi.org/10.1111/1758-2229.13137>

85.	Danilova OV, Oshkin IY, Belova SE *et al.* One Step Closer to Enigmatic USCα Methanotrophs: Isolation of a Methylocapsa-like Bacterium from a Subarctic Soil*.* *Microorganisms*, 2023. **11**: 2800. <https://doi.org/10.3390/microorganisms11112800>

86.	Seppey CV, Cabrol L, Thalasso F *et al.* Biogeography of microbial communities in high‐latitude ecosystems: Contrasting drivers for methanogens, methanotrophs and global prokaryotes*.* *Environmental Microbiology*, 2023. **25**: 3364-86. <https://doi.org/10.1111/1462-2920.16526>

87.	Danilova O, Belova S, Gagarinova I *et al.* Microbial community composition and methanotroph diversity of a subarctic wetland in Russia*.* *Microbiology*, 2016. **85**: 583-91. <https://doi.org/10.1134/s0026261716050039>

88.	Liebner S, Wagner D Abundance, distribution and potential activity of methane oxidizing bacteria in permafrost soils from the Lena Delta, Siberia*.* *Environmental microbiology*, 2007. **9**: 107-17. <https://doi.org/10.1111/j.1462-2920.2006.01120.x>

89.	Osudar R, Liebner S, Alawi M *et al.* Methane turnover and methanotrophic communities in arctic aquatic ecosystems of the Lena Delta, Northeast Siberia*.* *FEMS Microbiology Ecology*, 2016. **92**: fiw116. <https://doi.org/10.1093/femsec/fiw116>

90.	Liebner S, Rublack K, Stuehrmann T *et al.* Diversity of aerobic methanotrophic bacteria in a permafrost active layer soil of the Lena Delta, Siberia*.* *Microbial ecology*, 2009. **57**: 25-35. <https://doi.org/10.1007/s00248-008-9411-x>

91.	Khmelenina V, Makutina V, Kalyuzhnaya M *et al.* *Discovery of viable methanotrophic bacteria in permafrost sediments of northeast Siberia*. in *Doklady Biological Sciences*. 2002. Kluwer Academic Publishers-Plenum Publishers New York.

92.	Oshkin IY, Miroshnikov KK, Belova SE *et al.* Draft genome sequence of Methylovulum psychrotolerans Sph1T, an obligate methanotroph from low-temperature environments*.* *Genome Announcements*, 2018. **6**: 10-1128. <https://doi.org/10.1128/genomea.01488-17>

93.	Oshkin IY, Belova SE, Danilova OV *et al.* Methylovulum psychrotolerans sp. nov., a cold-adapted methanotroph from low-temperature terrestrial environments, and emended description of the genus Methylovulum*.* *International Journal of Systematic and Evolutionary Microbiology*, 2016. **66**: 2417-23. <https://doi.org/10.1099/ijsem.0.001046>

94.	Oshkin IY, Wegner C-E, Lüke C *et al.* Gammaproteobacterial methanotrophs dominate cold methane seeps in floodplains of West Siberian rivers*.* *Applied and environmental microbiology*, 2014. **80**: 5944-54. <https://doi.org/10.1128/AEM.01539-14>

95.	Yang S, Anthony SE, Jenrich M *et al.* Microbial methane cycling in sediments of Arctic thermokarst lagoons*.* *Global Change Biology*, 2023. **29**: 2714-31. <https://doi.org/10.1111/gcb.16649>

96.	Cabrol L, Thalasso F, Gandois L *et al.* Anaerobic oxidation of methane and associated microbiome in anoxic water of Northwestern Siberian lakes*.* *Science of the Total Environment*, 2020. **736**: 139588. <https://doi.org/10.1016/j.scitotenv.2020.139588>

97.	Dedysh S Exploring methanotroph diversity in acidic northern wetlands: molecular and cultivation-based studies*.* *Microbiology*, 2009. **78**: 655-69. <https://doi.org/10.1134/S0026261709060010>

98.	Dedysh SN, Derakshani M, Liesack W Detection and enumeration of methanotrophs in acidic Sphagnum peat by 16S rRNA fluorescence in situ hybridization, including the use of newly developed oligonucleotide probes for Methylocella palustris*.* *Applied and Environmental Microbiology*, 2001. **67**: 4850-7. <https://doi.org/10.1128/aem.67.10.4850-4857.2001>

99.	Dedysh SN, Pankratov TA, Belova SE *et al.* Phylogenetic analysis and in situ identification of bacteria community composition in an acidic Sphagnum peat bog*.* *Applied and Environmental Microbiology*, 2006. **72**: 2110-7. <https://doi.org/10.1128/aem.72.3.2110-2117.2006>

100.	Danilova OV, Kulichevskaya IS, Rozova ON *et al.* Methylomonas paludis sp. nov., the first acid-tolerant member of the genus Methylomonas, from an acidic wetland*.* *International journal of systematic and evolutionary microbiology*, 2013. **63**: 2282-9. <https://doi.org/10.1099/ijs.0.045658-0>

101.	Vorobev AV, Baani M, Doronina NV *et al.* Methyloferula stellata gen. nov., sp. nov., an acidophilic, obligately methanotrophic bacterium that possesses only a soluble methane monooxygenase*.* *International Journal of Systematic and Evolutionary Microbiology*, 2011. **61**: 2456-63. <https://doi.org/10.1099/ijs.0.028118-0>

102.	Dedysh SN, Panikov NS, Tiedje JM Acidophilic methanotrophic communities from Sphagnum peat bogs*.* *Applied and Environmental Microbiology*, 1998. **64**: 922-9. <https://doi.org/10.1128/AEM.64.3.922-929.1998>

103.	Dedysh SN, Khmelenina VN, Suzina NE *et al.* Methylocapsa acidiphila gen. nov., sp. nov., a novel methane-oxidizing and dinitrogen-fixing acidophilic bacterium from Sphagnum bog*.* *International Journal of Systematic and Evolutionary Microbiology*, 2002. **52**: 251-61. <https://doi.org/10.1099/00207713-52-1-251>

104.	Dedysh SN, Liesack W, Khmelenina VN *et al.* Methylocella palustris gen. nov., sp. nov., a new methane-oxidizing acidophilic bacterium from peat bogs, representing a novel subtype of serine-pathway methanotrophs*.* *International Journal of Systematic and Evolutionary Microbiology*, 2000. **50**: 955-69. <https://doi.org/10.1099/00207713-50-3-955>

105.	Dedysh S Methanotrophic bacteria of acidic Sphagnum peat bogs*.* *Microbiology*, 2002. **71**: 638-50. <https://doi.org/10.1023/A:1021467520274>

106.	Kox MA, Farhan Ul Haque M, van Alen TA *et al.* Complete genome sequence of the aerobic facultative methanotroph Methylocella tundrae strain T4*.* *Microbiology resource announcements*, 2019. **8**: 10-1128. <https://doi.org/10.1128/mra>. 00286-19

107.	Dedysh SN, Berestovskaya YY, Vasylieva LV *et al.* Methylocella tundrae sp. nov., a novel methanotrophic bacterium from acidic tundra peatlands*.* *International Journal of Systematic and Evolutionary Microbiology*, 2004. **54**: 151-6. <https://doi.org/10.1099/ijs.0.02805-0>

108.	Trotsenko YA, Khmelenina VN Aerobic methanotrophic bacteria of cold ecosystems*.* *FEMS Microbiology Ecology*, 2005. **53**: 15-26. <https://doi.org/10.1016/j.femsec.2005.02.010>

109.	Vecherskaya M, Galchenko V, Sokolova E *et al.* Activity and species composition of aerobic methanotrophic communities in tundra soils*.* *Current Microbiology*, 1993. **27**: 181-4. <https://doi.org/10.1007/bf01576018>

110.	Omel'Chenko M, Vasil'Eva L, Zavarzin G *et al.* A novel psychrophilic methanotroph of the genus Methylobacter*.* *Микробиология*, 1996. **65**: 384-9.

111.	Kalyuzhnaya M, Makutina V, Rusakova T *et al.* Methanotrophic communities in the soils of the Russian northern taiga and subarctic tundra*.* *Microbiology*, 2002. **71**: 227-33. <https://doi.org/10.1023/A:1015114724870>

112.	Berestovskaya YY, Vasil'eva L, Chestnykh O *et al.* Methanotrophs of the psychrophilic microbial community of the Russian arctic tundra*.* *Microbiology*, 2002. **71**: 460-6. <https://doi.org/10.1023/A:1019805929529>

113.	Sundh I, Borgå P, Nilsson M *et al.* Estimation of cell numbers of methanotrophic bacteria in boreal peatlands based on analysis of specific phospholipid fatty acids*.* *FEMS Microbiology Ecology*, 1995. **18**: 103-12. <https://doi.org/10.1111/j.1574-6941.1995.tb00167.x>

114.	Singleton CM, McCalley CK, Woodcroft BJ *et al.* Methanotrophy across a natural permafrost thaw environment*.* *The ISME journal*, 2018. **12**: 2544-58. <https://doi.org/s41396-018-0065-5>

115.	McDonald IR, Hall GH, Pickup RW *et al.* Methane oxidation potential and preliminary analysis of methanotrophs in blanket bog peat using molecular ecology techniques*.* *FEMS microbiology ecology*, 1996. **21**: 197-211. <https://doi.org/10.1111/j.1574-6941.1996.tb00347.x>

116.	McDonald IR, Murrell JC The particulate methane monooxygenase gene pmoA and its use as a functional gene probe for methanotrophs*.* *FEMS microbiology letters*, 1997. **156**: 205-10. <https://doi.org/10.1111/j.1574-6968.1997.tb12728.x>

117.	Chen Y, Dumont MG, McNamara NP *et al.* Diversity of the active methanotrophic community in acidic peatlands as assessed by mRNA and SIP‐PLFA analyses*.* *Environmental Microbiology*, 2008. **10**: 446-59. <https://doi.org/10.1111/j.1462-2920.2007.01466.x>

118.	Morris SA, Radajewski S, Willison TW *et al.* Identification of the Functionally Active Methanotroph Population in a Peat Soil Microcosm by Stable-Isotope Probing*.* *Applied and Environmental Microbiology*, 2002. **68**(3): 1446-1453. <https://doi.org/doi:10.1128/AEM.68.3.1446-1453.2002>

119.	He R, Wang J, Pohlman JW *et al.* Metabolic flexibility of aerobic methanotrophs under anoxic conditions in Arctic lake sediments*.* *The ISME Journal*, 2022. **16**: 78-90. <https://doi.org/s41396-021-01049-y>

120.	He R, Wooller MJ, Pohlman JW *et al.* Shifts in identity and activity of methanotrophs in arctic lake sediments in response to temperature changes*.* *Applied and environmental microbiology*, 2012. **78**: 4715-23. <https://doi.org/10.1128/AEM.00853-12>

121.	Esson KC, Lin X, Kumaresan D *et al.* Alpha-and gammaproteobacterial methanotrophs codominate the active methane-oxidizing communities in an acidic boreal peat bog*.* *Applied and environmental microbiology*, 2016. **82**: 2363-2371. <https://doi.org/10.1128/aem.03640-15>

122.	Gupta V, Smemo KA, Yavitt JB *et al.* Active methanotrophs in two contrasting North American peatland ecosystems revealed using DNA-SIP*.* *Microbial ecology*, 2012. **63**: 438-45. <https://doi.org/10.1007/s00248-011-9902-z>

123.	Costello AM, Lidstrom ME Molecular characterization of functional and phylogenetic genes from natural populations of methanotrophs in lake sediments*.* *Applied and environmental microbiology*, 1999. **65**: 5066-74. <https://doi.org/10.1128/aem.65.11.5066-5074.1999>

124.	Bechtold EK, Ellenbogen JB, Villa JA *et al.* Metabolic interactions underpinning high methane fluxes across terrestrial freshwater wetlands*.* *Nature Communications*, 2025. **16**: 944. <https://doi.org/10.1038/s41467-025-56133-0>

125.	Miller DN, Yavitt JB, Madsen EL *et al.* Methanotrophic activity, abundance, and diversity in forested swamp pools: spatiotemporal dynamics and influences on methane fluxes*.* *Geomicrobiology journal*, 2004. **21**: 257-71. <https://doi.org/10.1080/01490450490438766>

126.	Chen L, Li L, Zhang S *et al.* Anaerobic methane oxidation linked to Fe (III) reduction in a Candidatus Methanoperedens‐enriched consortium from the cold Zoige wetland at Tibetan Plateau*.* *Environmental microbiology*, 2022. **24**: 614-25. <https://doi.org/10.1111/1462-2920.15848>

127.	Xie F, Ma A, Zhou H *et al.* Niche differentiation of denitrifying anaerobic methane oxidizing bacteria and archaea leads to effective methane filtration in a Tibetan alpine wetland*.* *Environment International*, 2020. **140**: 105764. <https://doi.org/10.1016/j.envint.2020.105764>

128.	Zhang Y, Ma A, Liu W *et al.* The occurrence of putative nitric oxide dismutase (nod) in an alpine wetland with a new dominant subcluster and the potential ability for a methane sink*.* *Archaea*, 2018. **1**: 6201541. <https://doi.org/10.1155/2018/6201541>

129.	Luesken FA, Zhu B, van Alen TA *et al.* pmoA primers for detection of anaerobic methanotrophs*.* *Applied and environmental microbiology*, 2011. **77**: 3877-80. <https://doi.org/10.1128/aem.02960-10>

130.	Yang J, Jiang H, Wu G *et al.* Co-occurrence of nitrite-dependent anaerobic methane oxidizing and anaerobic ammonia oxidizing bacteria in two Qinghai-Tibetan saline lakes*.* *Frontiers of Earth Science*, 2012. **6**: 383-91. <https://doi.org/10.1007/s11707-012-0336-9>

131.	Zhu G, Zhou L, Wang Y *et al.* Biogeographical distribution of denitrifying anaerobic methane oxidizing bacteria in C hinese wetland ecosystems*.* *Environmental microbiology reports*, 2015. **7**: 128-38. <https://doi.org/10.1111/1758-2229.12214>

132.	Ren L, Wu X, Ma D *et al.* Nitrite-dependent anaerobic methane oxidation bacteria and potential in permafrost region of Daxing’an Mountains*.* *Applied Microbiology and Biotechnology*, 2022. **106**: 743-54. <https://doi.org/article/10.1007/s00253-021-11739-4>

133.	Li B, Tao Y, Mao Z *et al.* Iron oxides act as an alternative electron acceptor for aerobic methanotrophs in anoxic lake sediments*.* *Water Research*, 2023. **234**: 119833. <https://doi.org/10.1016/j.watres.2023.119833>

134.	Yang Y, Chen J, Li B *et al.* Anaerobic methane oxidation potential and bacteria in freshwater lakes: Seasonal changes and the influence of trophic status*.* *Systematic and Applied Microbiology*, 2018. **41**: 650-7. <https://doi.org/10.1016/j.syapm.2018.08.002>

135.	Weber HS, Habicht KS, Thamdrup B Anaerobic methanotrophic archaea of the ANME-2d cluster are active in a low-sulfate, iron-rich freshwater sediment*.* *Frontiers in Microbiology*, 2017. **8**: 619. <https://doi.org/10.3389/fmicb.2017.00619>

136.	Myllykangas J-P, Rissanen AJ, Hietanen S *et al.* Influence of electron acceptor availability and microbial community structure on sedimentary methane oxidation in a boreal estuary*.* *Biogeochemistry*, 2020. **148**: 291-309. <https://doi.org/10.1007/s10533-020-00660-z>

137.	Deutzmann JS, Schink B Anaerobic oxidation of methane in sediments of Lake Constance, an oligotrophic freshwater lake*.* *Applied and environmental microbiology*, 2011. **77**: 4429-36. <https://doi.org/10.1128/AEM.00340-11>

138.	Deutzmann JS, Stief P, Brandes J *et al.* Anaerobic methane oxidation coupled to denitrification is the dominant methane sink in a deep lake*.* *Proceedings of the National Academy of Sciences*, 2014. **111**: 18273-8. <https://doi.org/10.1073/pnas.1411617111>

139.	Zhu B, van Dijk G, Fritz C *et al.* Anaerobic oxidization of methane in a minerotrophic peatland: enrichment of nitrite-dependent methane-oxidizing bacteria*.* *Applied and environmental microbiology*, 2012. **78**: 8657-65. <https://doi.org/10.1128/AEM.02102-12>

140.	Ettwig KF, Butler MK, Le Paslier D *et al.* Nitrite-driven anaerobic methane oxidation by oxygenic bacteria*.* *Nature*, 2010. **464**: 543-8. <https://doi.org/10.1038/nature08883>

141.	Ettwig KF, van Alen T, van de Pas-Schoonen KT *et al.* Enrichment and molecular detection of denitrifying methanotrophic bacteria of the NC10 phylum*.* *Applied and environmental microbiology*, 2009. **75**: 3656-62. <https://doi.org/10.1128/aem.00067-09>

142.	Raghoebarsing AA, Pol A, Van de Pas-Schoonen KT *et al.* A microbial consortium couples anaerobic methane oxidation to denitrification*.* *Nature*, 2006. **440**: 918-21. <https://doi.org/articles/nature04617>

143.	Ettwig KF, Zhu B, Speth D *et al.* Archaea catalyze iron-dependent anaerobic oxidation of methane*.* *Proceedings of the National Academy of Sciences*, 2016. **113**: 12792-6. <https://doi.org/10.1073/pnas.1609534113>

144.	Ettwig KF, Shima S, Van De Pas‐Schoonen KT *et al.* Denitrifying bacteria anaerobically oxidize methane in the absence of Archaea*.* *Environmental microbiology*, 2008. **10**: 3164-73. <https://doi.org/10.1111/j.1462-2920.2008.01724.x>

145.	Winkel M, Mitzscherling J, Overduin PP *et al.* Anaerobic methanotrophic communities thrive in deep submarine permafrost*.* *Scientific reports*, 2018. **8**: 1291. <https://doi.org/10.1038/s41598-018-19505-9>

146.	Lomakina A, Pogodaeva T, Kalmychkov G *et al.* Diversity of NC10 bacteria and ANME-2d archaea in sediments of fault zones at Lake Baikal*.* *Diversity*, 2019. **12**: 10. <https://doi.org/10.3390/d12010010>

147.	Schubert CJ, Vazquez F, Lösekann-Behrens T *et al.* Evidence for anaerobic oxidation of methane in sediments of a freshwater system (Lago di Cadagno)*.* *FEMS microbiology ecology*, 2011. **76**: 26-38. <https://doi.org/10.1111/j.1574-6941.2010.01036.x>

148.	Su G, Zopfi J, Yao H *et al.* Manganese/iron‐supported sulfate‐dependent anaerobic oxidation of methane by archaea in lake sediments*.* *Limnology and Oceanography*, 2020. **65**: 863-75. <https://doi.org/10.1002/lno.11354>

149.	Echeveste Medrano MJ, Su G, Blattner LA *et al.* Methanotrophic Flexibility of 'Ca. Methanoperedens' and Its Interactions With Sulphate-Reducing Bacteria in the Sediment of Meromictic Lake Cadagno*.* *Environ Microbiol*, 2025. **27**: e70133. <https://doi.org/10.1111/1462-2920.70133>

150.	Smemo K, Yavitt J Anaerobic oxidation of methane: an underappreciated aspect of methane cycling in peatland ecosystems? *Biogeosciences*, 2011. **8**: 779-93. <https://doi.org/10.5194/bg-8-779-2011>

151.	Winkel M, Sepulveda-Jauregui A, Martinez-Cruz K *et al.* First evidence for cold-adapted anaerobic oxidation of methane in deep sediments of thermokarst lakes*.* *Environmental Research Communications*, 2019. **1**: 021002. <https://doi.org/10.1088/2515-7620/ab1042>

152.	Miller KE, Lai C-T, Dahlgren RA *et al.* Anaerobic methane oxidation in high-Arctic Alaskan peatlands as a significant control on net CH4 fluxes*.* *Soil Systems*, 2019. **3**: 7. <https://doi.org/10.3390/soilsystems3010007>
